# Efficacy of Blended Collaborative Care for Patients With Heart Failure and Comorbid Depression: A Randomized Clinical Trial

**DOI:** 10.1001/jamainternmed.2021.4978

**Published:** 2021-08-30

**Authors:** Bruce L. Rollman, Amy M. Anderson, Scott D. Rothenberger, Kaleab Z. Abebe, Ravi Ramani, Matthew F. Muldoon, John M. Jakicic, Bea Herbeck Belnap, Jordan F. Karp

**Affiliations:** 1 Division of General Internal Medicine, University of Pittsburgh School of Medicine, Pittsburgh, Pennsylvania; 2 Center for Behavioral Health, Media and Technology, University of Pittsburgh School of Medicine, Pittsburgh, Pennsylvania; 3 Center for Research on Health Care Data Center, University of Pittsburgh School of Medicine, Pittsburgh, Pennsylvania; 4 Heart and Vascular Institute, University of Pittsburgh School of Medicine, Pittsburgh, Pennsylvania; 5 Healthy Lifestyle Institute & Physical Activity and Weight Management Research Center, University of Pittsburgh, Pittsburgh, Pennsylvania; 6 Department of Psychosomatic Medicine and Psychotherapy, University of Göttingen Medical Center, Göttingen, Germany; 7 Department of Psychiatry, University of Pittsburgh School of Medicine, Pittsburgh, Pennsylvania; 8 Now with Department of Psychiatry, University of Arizona College of Medicine, Tucson

## Abstract

**Importance:**

Depression is often comorbid in patients with heart failure (HF) and is associated with worse clinical outcomes. However, depression generally goes unrecognized and untreated in this population.

**Objective:**

To determine whether a blended collaborative care program for treating both HF and depression can improve clinical outcomes more than collaborative care for HF only and physicians’ usual care (UC).

**Design, Setting, and Participants:**

This 3-arm, single-blind, randomized effectiveness trial recruited 756 participants with HF with reduced left ventricular ejection fraction (<45%) from 8 university-based and community hospitals in southwestern Pennsylvania between March 2014 and October 2017 and observed them until November 2018. Participants included 629 who screened positive for depression during hospitalization and 2 weeks postdischarge and 127 randomly sampled participants without depression to facilitate further comparisons. Key analyses were performed November 2018 to March 2019.

**Interventions:**

Separate physician-supervised nurse teams provided either 12 months of collaborative care for HF and depression (“blended” care) or collaborative care for HF only (enhanced UC [eUC]).

**Main Outcomes and Measures:**

The primary outcome was mental health–related quality of life (mHRQOL) as measured by the Mental Component Summary of the 12-item Short Form Health Survey (MCS-12). Secondary outcomes included mood, physical function, HF pharmacotherapy use, rehospitalizations, and mortality.

**Results:**

Of the 756 participants (mean [SD] age, 64.0 [13.0] years; 425 [56%] male), those with depression reported worse mHRQOL, mood, and physical function but were otherwise similar to those without depression (eg, mean left ventricular ejection fraction, 28%). At 12 months, blended care participants reported a 4.47-point improvement on the MCS-12 vs UC (95% CI, 1.65 to 7.28; *P* = .002), but similar scores as the eUC arm (1.12; 95% CI, −1.15 to 3.40; *P* = .33). Blended care participants also reported better mood than UC participants (Patient-Reported Outcomes Measurement Information System–Depression effect size, 0.47; 95% CI, 0.28 to 0.67) and eUC participants (0.24; 95% CI, 0.07 to 0.41), but physical function, HF pharmacotherapy use, rehospitalizations, and mortality were similar by both baseline depression and randomization status.

**Conclusions and Relevance:**

In this randomized clinical trial of patients with HF and depression, telephone-delivered blended collaborative care produced modest improvements in mHRQOL, the primary outcome, on the MCS-12 vs UC but not eUC. Although blended care did not differentially affect rehospitalization and mortality, it improved mood better than eUC and UC and thus may enable organized health care systems to provide effective first-line depression care to medically complex patients.

**Trial Registration:**

ClinicalTrials.gov Identifier: NCT02044211

## Key Points

**Question:**

Does blended collaborative care for heart failure and depression improve clinical outcomes more than collaborative care for heart failure alone or physicians’ usual care?

**Findings:**

In this randomized clinical trial of 629 patients with depression and heart failure, patients received either blended collaborative care, collaborative care for heart failure alone, or usual care. At 12 months, patients receiving blended care reported significantly better mental health–related quality of life (mHRQOL) and mood than those receiving usual care and better mood but not mHRQOL than those receiving care for heart failure alone; physical function, rehospitalizations, and mortality were similar among all 3 groups.

**Meaning:**

Blended collaborative care for heart failure and depression improved mHRQOL and mood more than usual care, and mood more than collaborative care for heart failure alone.

## Teaser

This randomized clinical trial assesses whether a blended collaborative care program for treating both heart failure and depression can improve clinical outcomes more than collaborative care for heart failure only and physicians’ usual care.

## Introduction

Heart failure (HF) affects approximately 6.2 million people in the US and is the leading cause for hospitalizations among Medicare patients.^1^ Depression is comorbid in 20% to 40% of patients with HF^2^ and associated with worse health-related quality of life (HRQOL),^3,4^ reduced adherence with recommended care,^5^ higher levels of health services utilization,^6,7,8^ and increased mortality.^9,10,11,12^ Yet despite the availability of proven-effective treatments, depression is often unrecognized and untreated in patients with HF.^13^

Several trials have examined the effect of treating depression as a means to improve clinical outcomes for patients with cardiovascular disease, but they reported mixed benefits on mood symptoms,^14,15,16,17,18,19^ and none reduced hospital admissions or mortality.^2^ Possible explanations include reliance on a single antidepressant that may be ineffective,^16,19^ small sample sizes,^14,17^ short follow-up duration, and enrollment of low-risk patients.^15,16,19^ Given the negative adverse effect of depression, interest remains in identifying effective treatments for depression that can be delivered at scale in routine HF practice.

One potential treatment strategy is collaborative care. Based on Wagner’s Chronic Care Model,^20^ it involves active follow-up by nonphysician care managers who support patients with the frequency of contacts necessary to educate them about their illness, impart evidence-based treatment recommendations, consider their prior treatment experiences and current preferences, and proactively monitor their responses to therapy and suggest adjustments in care under supervision of a physician. Trials have demonstrated the effectiveness of this model at improving patient outcomes for a variety of conditions, including HF,^21,22,23,24^ and we and others have demonstrated their clinical effectiveness^25,26,27,28,29^ and cost-effectiveness^30^ at treating depression in patients with cardiovascular disease. However, we are unaware of any collaborative care trial that focused on treating patients with depression and HF.^2,31^

Given the impracticality of separate collaborative care programs for HF and depression, integrated interventions that provide care for both conditions together may be an ideal means for organized systems of health care to deliver physical and mental health care at scale.^32,33^ Furthermore, evidence suggests that blended collaborative care may produce greater improvements in cardiovascular risk factors and mood symptoms^32^ than interventions focused solely on depression.^34,35^ To examine these issues in patients with HF, we conducted the Hopeful Heart Trial (https://www.hopefulheart.pitt.edu/) to test the effectiveness of a blended collaborative care strategy for treating both HF and depression. Hopeful Heart also included (1) a clinically relevant attention control arm to clarify whether any observed clinical improvements are due to our depression intervention rather than treatment for HF or patient expectations,^36^ and (2) a randomly selected cohort without depression to evaluate the influence of comorbid depression on the natural course of HF and the benefits derived from our study interventions.

## Methods

### Participants

Our full study protocol (Supplement 1; the statistical analysis plan is in Supplement 2) has been published^37^ and was approved by the University of Pittsburgh’s Institutional Review Board prior to the start of recruitment. Nurse-recruiters identified medically stable hospitalized patients aged 21 years and older with a left ventricular ejection fraction (LVEF) of 45% or less at 2 university-affiliated and 6 community Pittsburgh, Pennsylvania, area hospitals. After our study nurses obtained patients’ oral consent through a nurse or physician involved with their care, they confirmed the presence of New York Heart Association class II to IV symptoms and then administered the Patient Health Questionnaire (PHQ-2) depression screen.^38^

We classified the PHQ-2 screen as positive when a patient replied “yes” to at least 1 PHQ item and required that patients be medically stable and discharged home; have no current alcohol abuse, substance abuse, or bipolar disorder; not be in treatment with a mental health specialist or report active suicidality; have no communication barrier; and be mentally competent to provide consent.^39^ Upon verification, the nurse-recruiters obtained patients’ signed consent. Two weeks after hospital discharge, we telephoned patients to administer the PHQ-9^40^ and required they score 10 or higher to remain protocol eligible.

### Comparison Cohort Without Depression

We randomly sampled approximately 1 PHQ-2 screen-negative patient not using an antidepressant who met all other protocol eligibility criteria for every 4 randomized patients with depression, stratified by hospital and sex and oversampled by race. At the 2-week posthospitalization call, we required they score less than 5 on the PHQ-9 to remain protocol eligible.

### Assessments and Outcome Measures

Following confirmation of protocol eligibility, a research assessor administered our baseline telephone assessment. It included the Mental and Physical Component Summary of the 12-item Short Form Health Survey to measure mental HRQOL (mHRQOL) and physical HRQOL, respectively (MCS-12 and PCS-12)^41^; the 12-item Kansas City Cardiomyopathy Questionnaire (KCCQ-12)^42^; the 8-item fixed-length Patient-Reported Outcomes Measurement Information System–Depression (PROMIS-D) scale^43^; and the 17-item Hamilton Rating Scale for Depression (HRS-D).^44^ We also collected information on patients’ race and ethnicity, sex, address,^45^ and sociodemographic characteristics by self-report; and on comorbid medical conditions, medication use, and laboratory values by detailed medical record review.

Research assessors blinded to participants’ randomization assignments readministered our assessments by telephone 3, 6, and 12 months later and inquired about any hospitalizations since the previous assessment. When a hospitalization or death was reported, we obtained the relevant medical records and/or death certificates and 2 physicians independently reviewed and classified the event.

### Randomization Procedure

Following the baseline assessment, we randomized participants with depression using permuted blocks of varying size stratified by hospital type and patient sex prepared in advance by the study statistician (K.Z.A.) in a 2:2:1 ratio to either (1) collaborative care for HF and depression (“blended” care); (2) collaborative care for HF only (enhanced usual care [eUC]), or (3) physicians’ usual care (UC). To maintain our assessors’ blinding for telephone follow-up assessments, the project coordinator notified participants of their treatment assignment.

### Interventions

We organized our care managers into separate blended care and eUC teams of 2 to 3 nurses. To standardize our interventions and facilitate weekly case review sessions, we developed an electronic registry and checklist^46^ that systematically prompted each nurse to inquire about and document key aspects of HF care (eg, medications, blood pressure). The blended care registry also included prompts about depression care.^37^

### Enhanced Usual Care

Following randomization, care managers scheduled a 1-hour telephone call to review the patient’s cardiac history and medications to identify potential gaps in care; record HF symptoms, weight, and blood pressure; and impart basic HF education. Afterward, they scheduled 20- to 30-minute follow-up calls every 1 to 4 weeks for the first 3 months, and then monthly for the duration of our 12-month intervention. Based on participants’ clinical status, treatment preferences, and their case review discussions with the study cardiologist (R.R.), the care managers typically encouraged (1) adherence to guideline-recommended HF pharmacotherapy; (2) healthy lifestyle (eg, physical activity, tobacco cessation, and other HF self-care); (3) maintaining weight within a narrow range; and (4) keeping follow-up medical appointments.

### Blended Care

In addition to the eUC protocol, our blended care manager team provided collaborative care for depression based on our prior work.^47^ Specifically, they (1) inquired about participants’ psychiatric history; (2) provided basic psychoeducation; (3) assessed treatment preferences for depression; and (4) monitored mood symptoms with the PHQ-9. Following case review discussions with the study psychiatrist, they suggested treatment options that included initiation or adjustment of antidepressant pharmacotherapy prescribed by their primary care physician; adequate physical activity and sleep; “watchful waiting” for those with no history of depression and only mild elevations in mood (PHQ-9 score, 10-14); or referral to a mental health specialist if patients needed a higher level of care.

### Case Review

To minimize the potential for contamination between study arms, we held separate weekly case review meetings with our blended and eUC nurse care management teams and instructed staff to not discuss their patients with the other team. Sessions focused on newly randomized participants and those not doing well or with gaps in evidence-based HF care. Afterward, the care manager typically conveyed 1 to 3 treatment recommendations back to the patient and to their physician(s) via electronic medical record system, telephone, or fax. Patients and physicians could accept or reject our treatment recommendations and/or obtain care outside of the trial.

### Usual Care

For ethical reasons, we informed both UC participants and their primary care physicians about their baseline depression status. Participants receiving UC continued to receive care from their physicians, which could include referral to specialty cardiac or mental health care. However, we did not provide any treatment advice unless we detected suicidality or cardiac distress during a routine assessment.

### Data and Safety Monitoring

Whenever suicidal ideation was recorded in our electronic database, it automatically triggered a suicide risk management protocol that systematically guided staff to determine the frequency, chronicity, content, and threat level.^48^ Afterward, the staff member reviewed the information with the study psychiatrist, who determined the next steps. We also advised participants in possible cardiac distress to immediately telephone their physician, present to an emergency department, or call 911 as indicated. An independent data and safety monitoring board approved by our funding agency also monitored study progress and safety.

### Power and Sample Size

We designed Hopeful Heart to compare our blended care intervention to both UC and to eUC, the former to compare our findings with other collaborative care trials^32^ and the latter to control for the effects of HF collaborative care on mood.^36^ We selected the MCS-12 as our primary outcome measure because it is sensitive to changes in mood,^49^ clinically meaningful to patients, and permitted measurement of the effect of our interventions on both HF and depression. Because women with cardiovascular disease treated for depression may experience little benefit and perhaps harm vs usual care,^25,50,51^ we powered our trial to conduct within-sex analyses on our primary outcome measure.

Based on prior trials,^25,26,32^ we estimated that blended collaborative care could produce a clinically meaningful moderate 0.50 or greater effect size (ES) improvement on the MCS-12 vs UC at 12-month follow-up. Assuming an 80% 12-month assessment completion rate, intention-to-treat (ITT) analyses, and 2-sided α of .05, 125 blended care and 62 UC women (or men) with depression provided 80% power to detect within-sex 0.50 or greater ES improvement. We estimated that our blended care intervention would produce a smaller but still clinically meaningful 0.30 or greater ES improvement vs eUC. Using similar assumptions, 250 participants per arm provided 85% power to detect 0.30 or greater ES improvement and 80% power to detect 0.40 or greater ES within-sex improvements. We did not adjust for multiple comparisons in our power calculations.

### Statistical Analyses

We compared baseline sociodemographic, clinical, and functional status measures by baseline depression status using *t* tests for continuous data and χ^2^ tests for categorical data. Next we used linear mixed models^52^ with fixed effects for time, treatment arm, time-by-study arm, sex, hospital type, and a random effect to account for participant-level variability to estimate ITT changes in 12-month scores and ESs on all randomized participants with 95% CIs. We also performed a planned subgroup analysis of MCS-12 by sex and post hoc analysis of MCS-12 restricted to patients with a baseline PHQ-9 score of 15 or higher. Our ITT analyses used repeated measures linear mixed models to include all patients with data at any time point. To assess the effect of missing data, we compared baseline characteristics between those who did vs did not withdraw from the study, and we compared lost-to-follow-up rates between study arms. We assumed all data were missing-at-random and were robust to ignorable missingness assumptions.^53^ As a sensitivity analysis, we fit shared parameter models on the MCS-12, our primary outcome, and confirmed no association between time to dropout/mortality and scores over time.

We used Cox proportional hazard models adjusted for hospital recruitment site and sex to analyze time to first hospitalization and death by study arm and repeated these analyses within sex while adjusting for hospital recruitment site. Finally, we examined differences between treatment arms on various process of care measures (eg, pharmacotherapy), and applied similar ITT techniques to compare our cohorts with and without depression. All reported *P* values are 2-tailed with significance levels at *P* ≤ .05 with no adjustments for multiplicity, and all analyses were performed with STATA/SE, version 15.0 (StataCorp LLC). This study followed the Consolidated Standards of Reporting Trials (CONSORT) reporting guideline.

## Results

**Figure 1.**
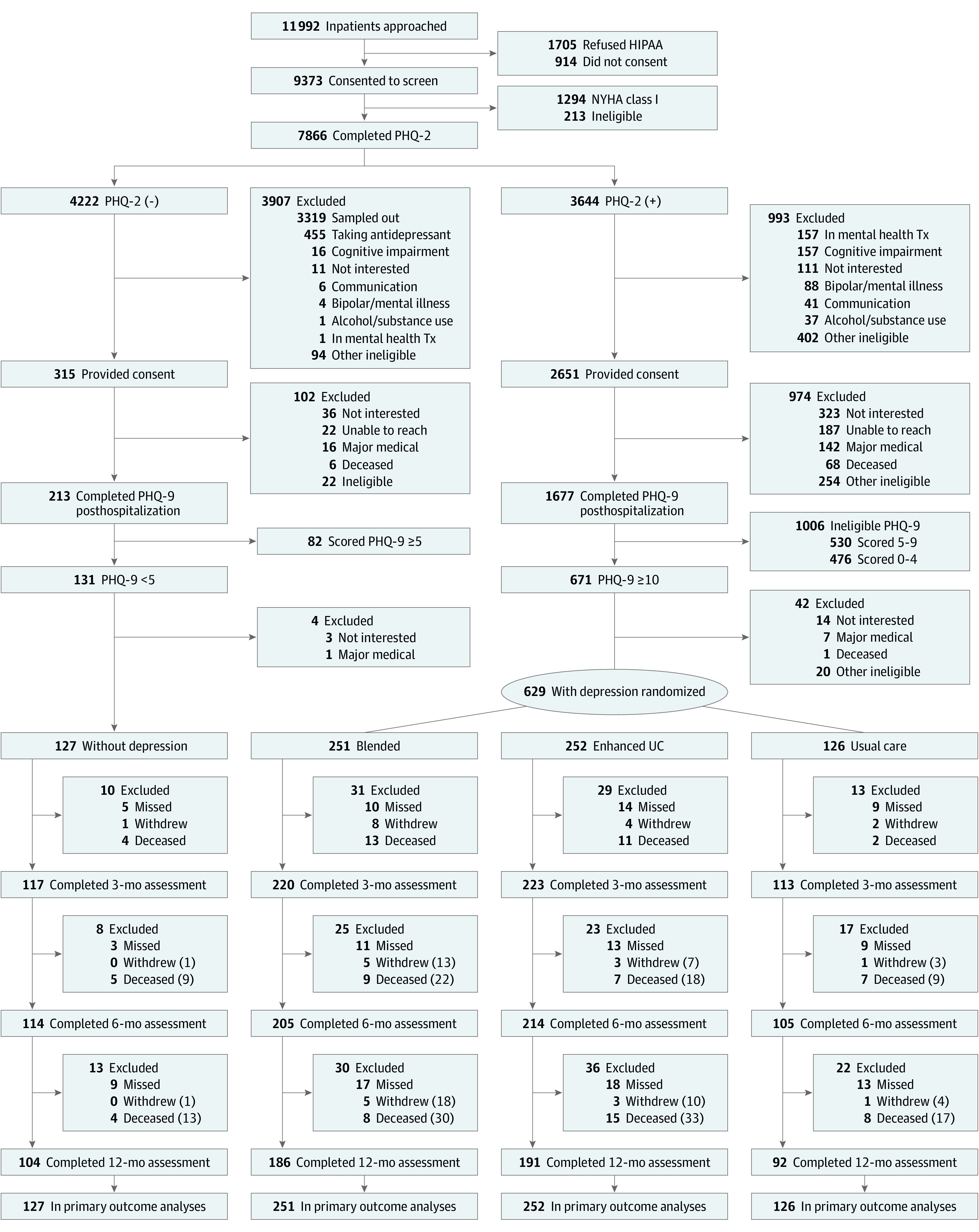
Flowchart of Screening, Enrollment, Randomization, and Follow-up^a^ ^a^Study participants who missed a 3- or 6-month follow-up assessment remained eligible to complete a later assessment. We display the cumulative number of withdrawn and deceased patients in parentheses. There were no differences in follow-up rates between randomized arms or by baseline depression status (eTable 1 in Supplement 3). HIPAA indicates Health Insurance Portability and Accountability Act; NYHA, New York Heart Association; PHQ, Patient Health Questionnaire; Tx, treatment; UC, usual care.

Of the 7866 hospitalized patients with HF who completed our PHQ-2 depression screening procedure between March 2014 and October 2017, 3644 (46%) screened positive and 2651 (73%) consented to enroll (Figure 1). At 2 weeks posthospitalization, 1677 (63%) remained eligible and completed the PHQ-9; 671 (40%) scored 10 or higher, and we randomized 629 who met all protocol eligibility criteria. Additionally, we enrolled 127 randomly selected patients with HF without depression for a total of 756 study participants. Rates of follow-up assessments were similar by baseline depression status and treatment assignment (eTable 1 in Supplement 3).

At baseline, participants with depression had similar sociodemographic and clinical characteristics by randomization status. However, compared with participants without depression, they were more likely to be White, have less formal education, be unemployed, smoke, and report lower HRQOL and physical functioning, despite having a similar mean (SD) LVEF (28% [9.4%]) and disease burden (Table 1).

**Table 1.** Baseline Sociodemographic and Clinical Characteristics by Randomization and Baseline Depression Status

Characteristic	No. (%)			
	With depression			Without depression (n = 127)
	Blended care (n = 251)	Enhanced usual care (n = 252)	Usual care (n = 126)	
Age, y, mean (SD)	63.4 (12.9)	64.6 (12.6)	62.2 (13.5)	65.7 (13.4)
Sex				
Female	110 (44)	108 (43)	55 (44)	58 (46)
Male	141 (56)	144 (57)	71 (56)	69 (54)
Race				
Black	60 (24)	57 (23)	27 (21)	45 (35)
White	186 (74)	190 (75)	97 (77)	78 (61)
Other	5 (2)	5 (2)	2 (2)	4 (3)
More than high school education	125 (50)	134 (53)	63 (50)	79 (62)
Married	105 (42)	98 (39)	59 (47)	56 (44)
Employed	39 (16)	21 (8)	15 (12)	24 (19)
Area Deprivation Index, mean (SD)^a^^,^^b^	68.0 (23.4) [n = 249]	66.3 (23.3) [n = 249]	64.5 (23.1) [n = 125]	67.3 (22.9) [n = 127]
NYHA class				
II	83 (33)	85 (34)	45 (36)	73 (57)
III	138 (55)	137 (54)	66 (52)	51 (40)
IV	30 (12)	30 (12)	15 (12)	3 (2)
LVEF, mean % (SD)	28.7 (9.2)	27 (9.4)	28.0 (9.5)	28.3 (8.3)
Hypertension	219 (87)	210 (84)	111 (88)	108 (85)
Diabetes	129 (52)	132 (52)	68 (54)	59 (46)
Hyperlipidemia	187 (75)	180 (71)	88 (70)	90 (71)
Current smoker	34 (14)	33 (13)	21 (17)	6 (5)
Myocardial infarction, prior	125 (50)	107 (42)	53 (42)	51 (40)
CABG surgery, prior	70 (28)	71 (28)	36 (29)	28 (22)
Implanted defibrillator	89 (36)	90 (36)	44 (35)	49 (39)
Kidney insufficiency	75 (30)	74 (29)	31 (25)	36 (29)
Medications				
ACE-I or ARB	154 (61)	148 (59)	73 (58)	76 (60)
β-Blocker	218 (87)	218 (87)	108 (86)	116 (91)
Diuretic	151 (60)	168 (67)	86 (68)	90 (71)
Statin	184 (73)	182 (72)	84 (67)	85 (67)
MCS-12, mean (SD)^b^^,^^c^	40.1 (10.8)	40.2 (10.8)	39.9 (11.8)	60.5 (4.9)
PCS-12, mean (SD)^b^^,^^c^	30.0 (10.0)	28.6 (9.2)	29.6 (10.0)	38.4 (11.2)
KCCQ-12, mean (SD)^b^^,^^c^	40.9 (19.7)	39.8 (20.3)	41.4 (19.8)	76.8 (17.7)
ESSI, mean (SD)^c^^,^^d^	26.4 (6.4)	26.0 (6.6)	26.9 (5.8)	30.9 (4.2)
PHQ-9, mean (SD)^e^^,^^f^	14.1 (3.4)	14.2 (3.7)	13.9 (3.9)	1.8 (1.3)
PHQ-9 ≥ 15	97 (39)	104 (41)	42 (33)	0
PROMIS-D, T-score mean (SD)^c^^,^^g^	60.3 (7.6)	60.1 (8.6)	60.1 (7.7)	41.8 (5.5)
HRS-D, mean (SD)^f^^,^^h^	16.8 (7.1)	17.0 (7.2)	17.4 (7.4)	2.0 (1.8)
Hospital recruitment type				
University	64 (26)	62 (25)	31 (25)	52 (41)
Community	140 (56)	413 (57)	71 (56)	62 (49)
Community underserved	47 (19)	47 (19)	24 (19)	13 (10)
Antidepressant medication				
Past year	109 (43)	105 (42)	55 (44)	9 (7)
Lifetime	140 (56)	137 (54)	72 (57)	24 (19)
Mental health specialist visit				
Past year	6 (2)	14 (6)	7 (6)	1 (1)
Lifetime	92 (37)	91 (36)	60 (48)	18 (14)

Abbreviations: ACE-I, angiotensin-converting enzyme inhibitor; ARB, angiotensin II receptor blocker; CABG, coronary artery bypass graft; ESSI, ENRICHD Social Support Index; HRS-D, Hamilton Rating Scale for Depression; KCCQ-12, Kansas City Cardiomyopathy Questionnaire; LVEF, left ventricular ejection fraction; NYHA, New York Heart Association; PHQ-9, 9-item Patient Health Questionnaire; PROMIS-D, Patient-Reported Outcomes Measurement Information System–Depression (fixed length, short form); MCS-12, Medical Outcomes Study Short Form Mental Health Composite Scale; PCS-12, Medical Outcomes Study Short Form Physical Health Composite Scale.

^a^ Higher scores indicate more deprivation over lower scores.

^b^ Range: 0-100.

^c^ Higher scores indicate better health.

^d^ Range: 8-34.

^e^ Range: 0-27.

^f^ Higher scores indicate worse health.

^g^ T-score range: 37.1-81.1.

^h^ Range: 0-53.

### Primary Outcome Measure

**Figure 2.**
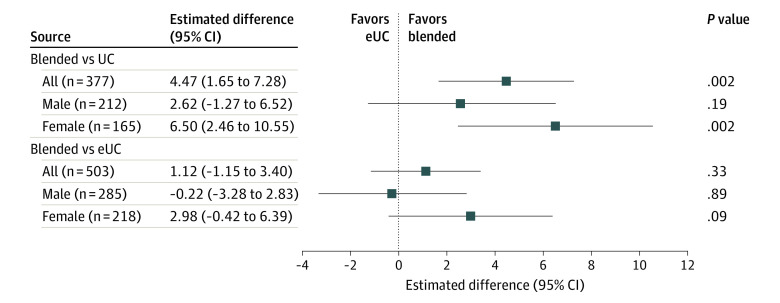
Twelve-Month Adjusted Differences in Mental Component Summary of the 12-Item Short Form Health Survey Scores for All Randomized Patients With Depression and by Sex Subgroup analysis by sex revealed no significant subgroup effect (*P* = .57 for 3-way interaction). eUC indicates enhanced usual care; UC, usual care.

At 12-month follow-up, patients with HF and depression randomized to blended care reported a 4.47-point improved MCS-12 score vs UC (95% CI, 1.65 to 7.28; *P* = .002) (Figure 2) (0.34 ES improvement; 95% CI, 0.13 to 0.56; *P* = .002) (eFigure 1 in Supplement 3). Within-sex analyses confirm the benefits of blended care vs UC for women (6.50-point improvement; 95% CI, 2.46 to 10.55; *P* = .002) but not men (2.62; 95% CI, −1.27 to 6.52; *P* = .19), and subgroup analyses by sex revealed no subgroup effect (*P* = .57 for 3-way interaction). Post hoc analyses restricted to patients with a baseline PHQ-9 score of 15 or higher identified a significant between-arm difference for blended vs UC patients (3.95-point improvement; 95% CI, 0.65 to 7.24; *P* = .02) but no significant within-sex effects.

**Figure 3.**
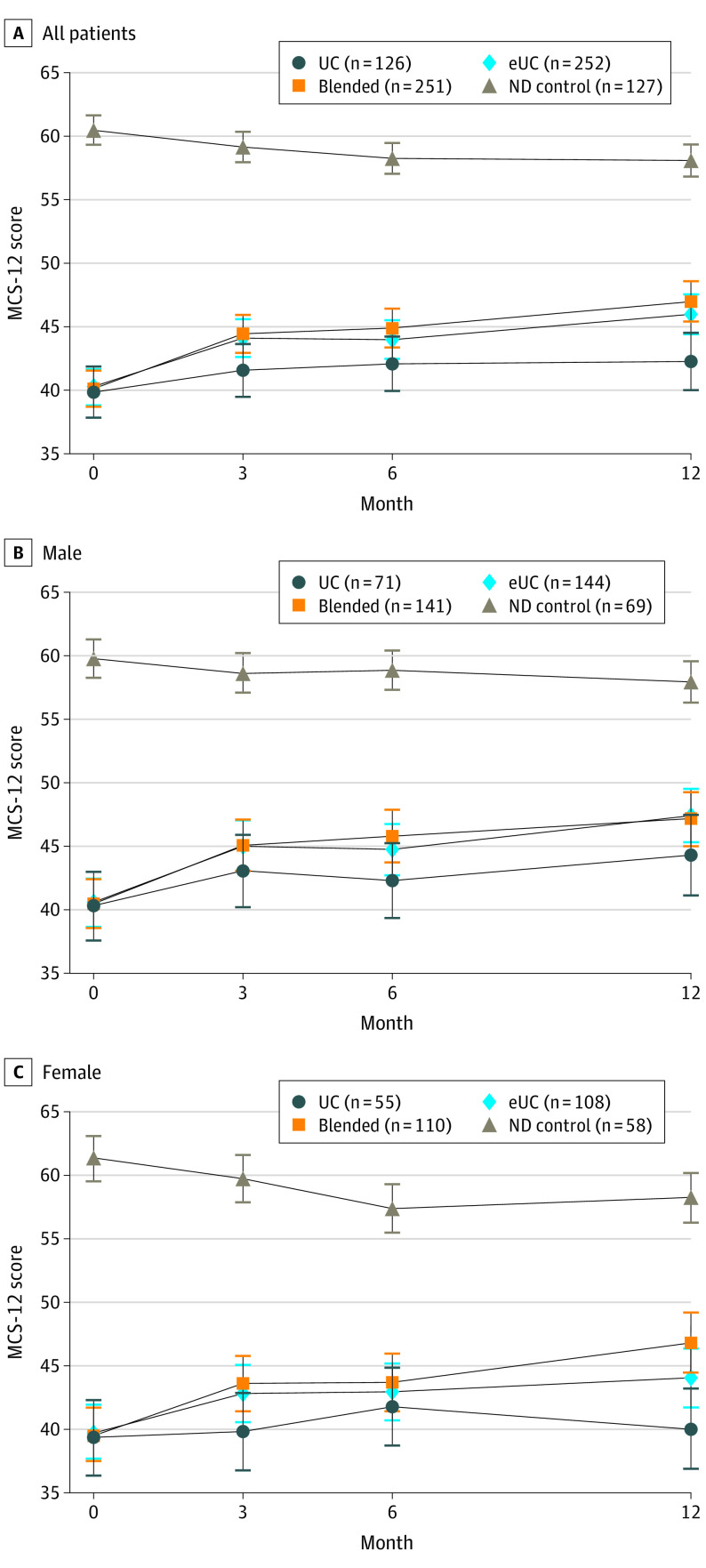
Estimated Mental Component Summary of the 12-Item Short Form Health Survey (MCS-12) Scores by Time Point for All Patients, Men, and Women Error bars indicate 95% CIs. eUC indicates enhanced usual care; ND, no depression; UC, usual care.

Comparing blended care to eUC patients, we found similar 12-month MCS-12 scores both among our full cohort (1.12-point improvement, 95% CI, −1.15 to 3.40; *P* = .33) and within each sex (Figure 2), and no significant sex subgroup effect. We display estimated mean MCS-12 scores at each assessment point for all study patients in Figure 3.

### Secondary Outcome Measures

#### Mood and Physical Function

At 12-month follow-up, patients receiving blended care reported improved mood on the PROMIS-D vs those receiving eUC (ES, 0.24; 95% CI, 0.07-0.41; *P* = .006) and UC (ES, 0.47; 95% CI, 0.28-0.67; *P* < .001), thus confirming the added benefits of collaborative care for patients with depression and HF (eFigure 1 in Supplement 3). Within-sex analyses confirm these blended vs UC findings for women (PROMIS-D, 0.65; 95% CI, 0.38-0.92; *P* < .001) and men (PROMIS-D, 0.31; 95% CI, 0.04-0.59; *P* = .03), and for women receiving blended care vs eUC (PROMIS-D, 0.41; 95% CI, 0.15-0.67; *P* = .002) but not men. However, analyzing HRS-D scores, we only found a differential improvement in mood symptoms for women receiving blended care vs UC (0.35; 95% CI, 0.02-0.67; *P* = .04). Patients receiving blended care also reported similar physical function (KCCQ-12) and physical HRQOL (PCS-12) vs both the UC and eUC arms (eFigure 1 in Supplement 3).

#### Care Process Measures

Although participants with depression were more likely to report taking a statin medication at 12 months, pharmacotherapy use was otherwise similar by baseline depression status. Also at 12 months, blended care and eUC participants had similar median numbers of nurse care manager contacts and mental health specialty visits and rates of antidepressant and HF pharmacotherapy use and adjustments (Table 2).

**Table 2.** Twelve-Month Care Processes and Health Services Use Following Randomization

Care process/health service	With depression			Without depression (n = 127)	*P* value^a^
	Blended care (n = 251)	Enhanced usual care (n = 252)	Usual care (n = 126)		
**Mental health care**					
Care manager contacts, median (range), mo					
3	3 (0-7)	3 (0-15)	NA	NA	NA
6	5 (0-12)	5 (0-23)	NA	NA	NA
12	9 (0-17)	9 (0-33)	NA	NA	NA
SSRI/SNRI use, No. (%)^b^					
Randomization	75 (30)	79 (31)	46 (37)	0	<.001
12 mo	66 (36)	68 (35)	39 (42)	0	<.001
Mental health specialty visits, median (range), mo					
6	0 (0-2)	0 (0-1)	0 (0-0)	0 (0-0)	.34
12	0 (0-2)	0 (0-1)	0 (0-0)	0 (0-0)	.19
**HF pharmacotherapy** ^b^					
ACE-I/ARB, No. (%)					
Randomization	145 (58)	142 (56)	71 (56)	72 (57)	.96
12 mo	106 (57)	106 (55)	48 (52)	62 (60)	.44
β-Blocker, No. (%)					
Randomization	215 (86)	213 (85)	108 (86)	113 (89)	.27
12 mo	156 (84)	156 (81)	79 (86)	87 (84)	.94
Diuretic, No. (%)					
Randomization	153 (61)	165 (65)	83 (66)	89 (70)	.17
12 mo	119 (64)	117 (61)	58 (63)	69 (66)	.48
Statin, No. (%)					
Randomization	180 (72)	174 (69)	85 (67)	82 (65)	.25
12 mo	129 (70)	143 (74)	64 (70)	61 (59)	.01
Medications: started or stopped by 12 mo, No (%)^c^					
SSRI/SNRI	29 (16)	20 (10)	8 (9)	NA	.16
ACE-I/ARB	37 (20)	38 (20)	15 (16)	NA	.73
β-Blocker	20 (11)	25 (13)	12 (13)	NA	.77
Diuretic	24 (13)	37 (19)	18 (20)	NA	.19
Statin	14 (8)	21 (11)	9 (10)	NA	.52
**Rehospitalizations** ^d^					
All cause					
6 mo, median (range)	0 (0-7)	0 (0-6)	0 (0-6)	0 (0-5)	.23
12 mo, median (range)	1 (0-13)	1 (0-9)	1 (0-14)	1 (0-11)	.39
Incident rate/person-year (95% CI)	0.99 (0.84-1.17)	1.10 (0.94-1.29)	0.93 (0.74-1.18)	0.85 (0.90-1.09)	.22
Cardiovascular related					
6 mo, median (range)	0 (0-5)	0 (0-4)	0 (0-5)	0 (0-3)	.77
12 mo, median (range)	0 (0-8)	0 (0-6)	0 (0-12)	0 (0-10)	.82
Incident rate/person-year (95% CI)	0.62 (0.51-0.75)	0.62 (0.51-0.75)	0.55 (0.41-0.73)	0.51 (0.38-0.68)	.35

Abbreviations: ACE-I, angiotensin-converting enzyme inhibitor; ARB, angiotensin receptor blocker; HF, heart failure; NA, not applicable; SNRI, serotonin-norepinephrine reuptake inhibitor; SSRI, selective serotonin reuptake inhibitor.

^a^ Depression vs no depression unless otherwise noted.

^b^ Data from medical record abstraction.

^c^ Among those who completed 12-month assessment, number of patients per randomized arm that started or stopped a medication between baseline and 12-month. For blended care, n = 186; enhanced usual care, n = 191; usual care, n = 92. *P* value compares proportions of changes in medication across randomized arms.

^d^ See eTable 3 in Supplement 3 for further details.

#### Rehospitalizations and Mortality

By November 1, 2018, after the last 12-month follow-up contact, we identified 1145 rehospitalizations (533 cardiovascular-related; eTable 2 in Supplement 3). We ascertained vital status on all 756 participants (100%) and identified 98 deaths, all from nonsuicidal causes (79 cardiovascular-related; eTable 3 in Supplement 3), and found no serious or unexpected adverse events.

We observed similar patterns for the incidence of all-cause and cardiovascular readmissions (eFigure 2 in Supplement 3) even after calculating incidence rate per person-year to adjust for multiple readmissions within a single patient (all-cause readmissions: 61.2% blended care; 53.1% eUC; 58.2% UC; 56.5% controls without depression) (eTable 2 in Supplement 3). The cumulative incidence of 12-month all-cause and cardiovascular mortality was similar by treatment assignment (all-cause: 13.4% blended care, 10.2% eUC, 13.2% UC), within sex, and by baseline depression status (14.1% controls without depression) (eFigure 3 in Supplement 3).

## Discussion

At 12-month follow-up, telephone-delivered blended collaborative care for treating both HF and depression improved mHRQOL and mood symptoms on the PROMIS-D more than physicians’ UC, and improved PROMIS-D scores but not mHRQOL compared with collaborative care for HF alone. However, it did not differentially improve HRS-D mood symptoms, physical HRQOL, or function; increase use of HF or antidepressant pharmacotherapy; or affect rates of hospital readmissions and mortality between any of our comparison groups (eUC, UC, or controls without depression).

To our knowledge, Hopeful Heart is the first trial to apply a blended collaborative care approach to treat depression in patients with HF. Our blended intervention generated similar improvements in mHRQOL vs UC as reported in our collaborative care trial for post-CABG depression (SF-36 MCS ES: 0.30; 95% CI, 0.17 to 0.52)^25^ and by other investigators.^24,26,27,28,29^ To our knowledge, Hopeful Heart is also the first collaborative care trial for depression that included a potent and clinically relevant attention control arm, thus supporting the effectiveness of this strategy at improving mood.^36^

Owing to the mutually exacerbating effects of depression on comorbid medical illness,^2^ we speculated that our blended care intervention would generate larger improvements in mood symptoms and improve HF outcomes more than if we focused solely on depression. Supporting this hypothesis, the TEAMcare trial by Katon et al^32^ of blended collaborative care for depression and poorly controlled diabetes reported large improvements in mood (0.67 ES) and diabetes control vs UC. Therefore, we were surprised to find similar usage of guideline-recommended HF pharmacotherapy regardless of treatment assignment. As HF pharmacotherapy use was also similar by baseline depression status (except statin use), our findings may reflect ceiling effects (84% β-blocker use), medication intolerance or contraindication (29% baseline kidney insufficiency), difficulty affording medications (13% employed), or clinical inertia.^54^ We frequently encountered patients challenged to follow our treatment recommendations owing to out-of-pocket costs for medical services and medications, lack of transportation to medical appointments, and inadequate access to heart-healthy food. These factors could have contributed to the similarities in rehospitalization and mortality rates among all study arms. Thus, we encourage new approaches to treat HF and other medically complex patients that address these barriers^55,56^ and build on our efforts.^57^

The external and internal validity of our findings is supported by recruitment of patients from community and underserved hospitals, application of standardized enrollment criteria, randomization, blinded outcomes assessments, inclusion of both a clinically relevant attention control resembling the HF care provided by many organized health care delivery systems and a UC control, and centralized telephone-delivered intervention. We also included a randomly selected cohort of patients with HF without depression to evaluate the effect of comorbid depression and control for changes in medical care and insurance coverage^58^ over the duration of our study.

### Limitations

Our study has several limitations. First, we limited enrollment to hospitalized patients with confirmed systolic dysfunction and excluded those with preserved LVEF to ensure that all study patients had HF and not another condition whose symptoms overlap (eg, emphysema). Second, we enrolled patients hospitalized for new or recurrent episodes of HF decompensation and other medical conditions that could have influenced their risks of rehospitalization and adverse cardiac events and need to adjust their HF medications. Third, we relied on the 2-step PHQ rather than a psychiatric interview to classify patients as having depression.^59^ Still, an American Heart Association science advisory recommends the PHQ because it can be administered by non–mental health professionals,^60^ and we rescreened patients following hospital discharge to confirm the persistence of mood symptoms. Finally, there was the potential for treatment contamination, as community clinicians may have cared for patients in different study arms and our single study cardiologist participated in both blended and eUC case review sessions. While a cluster randomized clinical trial involving separate teams of study cardiologists could minimize contamination, this study design would require a much larger sample size and greater expense to conduct.

## Conclusions

Despite the modest improvements of telephone-delivered blended collaborative care for HF and depression on clinical outcomes vs physicians’ UC and collaborative care for HF alone in this randomized clinical trial, blended strategies such as ours may enable organized health care systems to provide effective first-line care for comorbid depression and other mental health conditions at scale to medically complex patients. Further research is needed to understand the cost-effectiveness of this approach and test adaptations that help patients overcome barriers to recommended treatments.

## Notes

Supplement 1. Trial Protocol

Supplement 2. Statistical Analysis Plan

Supplement 3. **eFigure 1.** 12-Month effect sizes for all randomized depressed patients and by gender. **eFigure 2.** 12-Month Readmissions: (A) All-Cause; and (B) Cardiovascular-Related. **eFigure 3.** 12-Month Mortality: (A) All-Cause; and (B) Cardiovascular-Related. **eTable 1.** Significance test for follow up rates between randomized arms and between depressed vs non-depressed. **eTable 2.** Readmission incidence rate per person year, All-Cause and Cardiovascular. **eTable 3.** Mortality incidence rate per person year, All-Cause and Cardiovascular.

Supplement 4. Data Sharing Statement
