# Supplementary material for: Efficacy of Blended Collaborative Care for Patients With Heart Failure and Comorbid Depression: A Randomized Clinical Trial
Source: JAMA Intern Med. 2021 Aug 30;181(10):1369–80. doi: 10.1001/jamainternmed.2021.4978 (PMC8406216; doi:10.1001/jamainternmed.2021.4978)
Supplement: Supplement 1. — Trial Protocol [file jamainternmed-e214978-s001.pdf]

Title of Research Study: Blended Collaborative Care for Heart Failure and Co-Morbid Depression

Triage Section

T1.0 Select the type of application:

New Research Study

T2.0 Is the proposed research study limited to the inclusion of deceased individuals?

No. The review and approval of proposed innovative practices are not subject to IRB review and approval. The introduction of innovative procedures or therapies into clinical practice (i.e., independent of a research activity approved by the IRB) should be reviewed with the applicable department chairperson and the UPMC Technology Assessment Committee/Innovative Practices Sub-Committee prior to their implementation. The contact person is Mary Gardner at 412-647-6883.

T2.1 Are any research activities being conducted at the VA Pittsburgh Healthcare System or with VA funds?

No.

Respond to the following questions to determine the IRB-of-record:

- **Research is conducted using only VA records and/or subjects recruited thru the VA:**
- **University or UPMC facilities are not engaged in research:**
- **University or UPMC funds are not expended in direct support of research:**

If all true, then the VA is the IRB-of-record and UPitt IRB review is not required. If all false, only UPitt IRB review is required. Otherwise, dual review from both the VA and UPitt IRB is required.

Quality assurance projects are not subject to IRB review and approval. UPMC has adopted an oversight process that requires the submission of all quality assurance projects for review. At UPMC, submissions are reviewed by the Total Quality Council. The contact person is Juliet Jegasothy at 412-612-3304.

Research studies that are limited to the inclusion of deceased individuals are not subject to IRB review and approval. Research performed on individuals who have been declared legally dead and/or research involving the collection of tissues from deceased individuals is not subject prior review and approval by the University of Pittsburgh IRB.

There are, however, ethical issues associated with research conducted on or involving deceased individuals. To address these ethical issues, all University faculty who desire to perform research on or involving deceased individuals must submit a project application for review and approval by the Committee for Oversight of Research and Clinical Training Involving the Dead Research Involving the Dead (CORID). Note that, as per UPMC policies, research involving the medical records of deceased individuals is subject to obtaining the written consent of the decedents’ next-of-kin or the executors of the decedents’ estates.

For studies that include BOTH living and deceased subjects, IRB review and approval is required.

T3.0 What is the anticipated risk to the research participants?

Minimal Risk

T3.1 Why do you feel that all aspects of this research study, including screening and follow-up, involve no more than minimal risk to the research subjects?

This study is minimal risk because our protocol does not involve the use of any investigational new drugs, devices, or any other treatments. Any medications that patients may receive for their depression, anxiety, or caridac condition will be prescribed by their physicians as we (the study) will neither prescribe nor provide any medications. The risks involved in this study are: 1) the potential for a breach of confidentiality; 2) the possibility that patients may experience discomfort discussing disturbing symptoms and/or psychosocial concerns with a study care manager; and 3) that a patient may be falsely labeled as having a mood disorder and offered treatment for it. We will take multiple steps to ensure the confidentiality of patients as described in section D4b of our NHLBI grant application (e.g., only select members of our research team will have electronic access to specific folders in secured servers that contain de-identified patient data; identified data will be stored in a separate table with only the database manager and project coordinator having access; paper consent forms and other primary data sources will be stored in locked files at the Center for Research on Health Care where the investigators’ offices and our Data Center are located with access provided only to designated research personnel to ensure strict confidentiality; and no personal information concerning study subjects will be released without their written consent, with the exception of active suicidality which will be released only to the subject’s personal physician(s) or an emergency team, and no patient will be identified in any description or publication of this research).

T4.0 Does the proposed study qualify for 'exempt' IRB review or for a determination of either 'not research' or 'no human subject' involvement?

No

T5.0 Does the proposed research study qualify for 'expedited' IRB review status?

No

<u>**Cover Sheet Section**</u>

CS1.0 What is the reason for this submission?

New Research Protocol Submission

CS1.1 Has this research study been approved previously by the University of Pittsburgh IRB?

No

CS1.1.1 Has this research study (or a substantially similar research study) been previously disapproved by the University of Pittsburgh IRB or, to your knowledge, by any other IRB?

No

CS2.0 Title of Research Study:

Blended Collaborative Care for Heart Failure and Co-Morbid Depression

CS2.1 Research Protocol Abstract:

Heart failure (HF) is an important public health problem that affects approximately 6.6 million Americans. Despite improvements in cardiac care, it remains the leading cause for hospitalization among Medicare patients and the only major cardiovascular disease whose mortality rate has remained essentially unchanged over the past decade. This failure to improve HF outcomes may be due, in part, to unrecognized and/or inadequately treated depression that is highly prevalent in HF patients. Yet while new HF treatment guidelines advocate routine screening for depression, this recommendation is unlikely to be widely adopted without trial evidence that depression care improves outcomes and efficient methods to provide it. “Collaborative care” strategies are being increasingly utilized to improve care for HF and other chronic medical conditions, and we recently demonstrated its clinical and cost-effectiveness at treating depression following coronary artery bypass graft surgery. Yet it may be impractical for health care delivery systems to support separate treatment programs for HF and depression. Thus we are encouraged by emerging evidence indicating “blended” collaborative care strategies that target both psychiatric and physical conditions produce greater improvements in mood symptoms and control of cardiovascular risk factors than programs focused solely on depression to propose testing a novel adaptation that could be provided in routine care. The Specific Aims of this Project are to: (1) evaluate the effectiveness of a telephone-delivered “blended” collaborative care intervention for treating HF and depression that could be adopted into routine clinical practice if proven effective; and (2) advance our understanding of the moderators and mediators of depression treatment on clinical outcomes. We will screen hospitalized patients with systolic HF for depression, and then randomize 625 who screen positive and have at least a moderately elevated level of depressive symptoms at two-weeks following hospital discharge to either: (1) collaborative care for treating both HF and depression (“blended”); (2) collaborative care for treating HF alone (enhanced usual care (eUC)); or (3) their doctors’ “usual care” (UC). Additionally, we will enroll 125 non-depressed HF patients to better evaluate the benefits derived from treating depression (total N=750). Our co-primary hypotheses will test whether “blended” collaborative care can produce at 12-months follow-up a: (A) 0.50 effect size (ES) or greater improvement in health-related quality of life (HRQoL) vs. UC; and (B) 0.30 ES or greater improvement in HRQoL vs. eUC. Secondary hypotheses will evaluate the effects of our “blended” intervention on mood, functional status, adherence with guideline-consistent care, incidence of cardiovascular events, health care utilization, and costs. Improving chronic illness care for medically complex patients is one of the major challenges facing medicine today. We propose to test the effectiveness of an innovative, efficient, scalable, and sustainable intervention that could transform the way HF and other cardiovascular disorders.

CS3.0 Name of the Principal Investigator:

Bruce Rollman

CS3.1 Affiliation of Principal Investigator:
UPitt faculty member

CS3.2 Address of Principal Investigator:
230 Mckee Place, Suite 600
Pittsburgh, PA 15213

CS3.3 Recorded Primary Affiliation of the Principal Investigator:
U of Pgh | School of Medicine

CS3.4 Identify the School, Department, Division or Center which is responsible for oversight of this research study:
U of Pgh | School of Medicine | Medicine | General Internal Medicine

CS3.5 Telephone Number of Principal Investigator:
412-692-4850

CS3.6 Recorded Current E-mail Address of Principal Investigator to which all notifications will be sent:
rollmanbl@upmc.edu

CS3.7 Fax Number:
412-692-4838

CS3.8 Does this study include any personnel from Carnegie Mellon University, and/or use any CMU resources or facilities (e.g., Scientific Imaging and Brain Research Center (SIBR)?
No

CS4.0 List of Co-Investigators:

Last	First	Organization
Champion	Hunter	U of Pgh | School of Medicine | Medicine
Jakicic	John	U of Pgh | School of Education | Health Physical and Recreation Education
Karp	Jordan	U of Pgh | School of Medicine | Psychiatry
Mazumdar	Sati	U of Pgh | Graduate School of Public Health | Biostatistics
Muldoon	Matthew	U of Pgh | School of Medicine | Medicine
Reynolds	Charles	U of Pgh | School of Medicine | Psychiatry
Smith	Kenneth	U of Pgh | School of Medicine | Medicine | General Internal Medicine

CS5.0 Name of Primary Research Coordinator:
Bea Herbeck-Belnap

CS5.1 Address of Primary Research Coordinator:
230 McKee Place, Suite 600
Pittsburgh, PA 15213

CS5.2 Telephone Number of Primary Research Coordinator:
412-692-2666

CS6.0 Name of Secondary Research Coordinator
N/A

CS7.0 Will this research study use any Pediatric PittNet or Clinical and Translational Research Center (CTRC) resources?
No

CS8.0 Select the entity responsible for scientific review.
External Scientific Review Completed – The scientific merit of this research protocol has been confirmed by an external scientific review committee as a condition of funding.

CS9.0 Does this research study involve the administration of an investigational drug or an FDA-approved drug that will be used for research purposes?
No

CS10.0 Is this research study being conducted under a University of Pittsburgh-based, sponsor-investigator IND or IDE application?
No

CS11.0 Is this research study supported in whole or in part by industry? This includes the provision of products (drugs or devices).
No

Is this a multi-centered study?
No

CS12.0 Does this research study involve 1) the use or evaluation of an investigational radioactive drug or an investigational radiation-emitting device; 2) an experimental intervention that involves radiation exposure; or 3) any standard radiation-emitting procedures (e.g., chest X-rays, CT scans, FDG-PET studies) that are being performed for screening and/or follow-up purposes at a substantially greater frequency than what would be encountered in routine clinical practice? (Note: “investigational” means not currently approved by the FDA for commercial marketing; “experimental intervention” means the drug or procedure being specifically evaluated in the research study).
No

CS13.0 Does this research study involve the deliberate transfer of recombinant DNA (rDNA) or DNA or RNA derived from rDNA into human subjects?

No

CS14.0 Are you using UPMC facilities and/or UPMC patients during the conduct of your research study?

Yes (Research Fiscal Review Form attached in IRB form)

CS15.0 Indicate the sites where research activities will be performed and/or private information will be obtained.

UPMC

Sites:
UPMC Presbyterian
UPMC Mercy
UPMC Shadyside
UPMC St. Margaret
UPMC Passavant
UPMC McKeesport

CS15.1 Have you, Bruce Rollman , verified that all members of the research team have the appropriate expertise, credentials, and if applicable, hospital privileges to perform those research procedures that are their responsibility as outlined in the IRB protocol?

Yes

CS15.2 Describe the availability of resources and the adequacy of the facilities to conduct this study:

The six UPMC-affiliated hospitals named in CS 15 annually care for the largest numbers of heart failure inpatients in the UPMC system (>2,500 unique heart failure patients discharged alive in CY 2010; see Table 1 from grant application). We estimate these hospitals will provide the necessary volume of patients we will need to screen over a 36-month period to achieve our target sample size (Figure 2).These hospitals are also located within 12 miles of the PI and his investigative team on the main Oakland medical campus of the University of Pittsburgh.

CS16.0 Special Research Subject Populations

None

CS17.0 Does your research involve the use of ANY human stem cells?

No

1.0 – Objective, Aims, Background and Significance

1.1 Objective: What is the overall purpose of this research study?

The purpose of the study is to compare the effectiveness of a “blended” telephone-delivered collaborative care intervention for treating both heart failure (HF) and depression ("Blended") to: (1) collaborative care for heart failure-alone (“enhanced usual care”; eUC); and (2) doctors’ “usual care” for depression (UC). If proven effective and cost-effective, the potentially more powerful, scalable, efficient “blended” care approach for treating HF and co-morbid depression could have profound implications for improving chronic illness care and stimulate development of “blended” interventions for treating other clusters of related medical conditions.

1.2 Specific Aims: List the goals of the proposed study.

Specific Aims are to: (1) evaluate the effectiveness of a telephone-delivered “blended” collaborative care intervention for treating both depression and HF that could be readily adopted into routine clinical practice if proven effective; and (2) advance our understanding of the moderators and mediators of depression treatment on clinical outcomes.

We propose to screen hospitalized adults with HF for depression and then randomize 625 protocol-eligible patients with a cardiac ejection fraction (EF) ≤ 40%, New York Heart Association (NYHA) class II-IV symptoms, and who have at least a moderately elevated level of depressive symptoms (PHQ-9 ≥ 10) at two-weeks following hospital discharge to a 12-month course of either: (1) Collaborative care for treating both HF and depression (“blended”); (2) Collaborative care for treating HF alone (enhanced usual care (eUC)); or (3) Their doctors’ “usual care” for treating HF and depression (UC).

Additionally, we will enroll 125 non-depressed HF patients to better evaluate the benefits derived from treating depression and, together with UC, control for any secular changes in the management of HF as elements of the 2010 Affordable Care Act are phased-in (Total N=750). Our co-primary hypotheses will test whether “blended” collaborative care can produce at 12-months follow-up a: (A) moderate 0.50 effect size (ES) or greater improvement in HRQoL vs. UC; and (B) clinically meaningful 0.30 ES or greater improvement in HRQoL vs. eUC. Our secondary hypotheses will evaluate in comparisons with eUC and UC the effects of our “blended” intervention on mood, functional status, delivery of and adherence with guideline-consistent care, incidence of cardiovascular events, health care utilization, and costs.

Though not explicit objectives of this application, the proposed trial will also serve as the basis for ancillary studies to examine the genomic moderation of treatment effects and such mediating mechanisms as blood pressure control, sleep quality, and chronic inflammation to further advance our understanding of how depression and its treatment affects clinical outcomes.

1.3 Background: Briefly describe findings or observations that provide the background leading to this proposal.

HF is an important and growing public health problem that affects over 6.6 million Americans with over 660,000 newly diagnosed cases, 280,000 deaths, and $39 billion in direct and indirect costs yearly. Despite improvements in cardiac care in recent years, including deployment of various disease management programs to improve delivery of guideline-recommended pharmacotherapy and promote patient self-management, HF remains the leading cause for hospitalization among Medicare patients and the only major cardiovascular disease whose mortality rate has remained essentially unchanged over the past decade. This failure to improve HF outcomes may be due, in part, to unrecognized and/or inadequately treated depression.

A meta-analysis of 27 studies identified a 16-38% prevalence of depression among hospitalized patients with HF depending on the method of diagnosis, gender, and HF severity, and strong evidence links depression to reduced HRQoL, higher levels of treatment costs and health services utilization, and increased morbidity and mortality independent of disease severity. In recognition, current HF treatment guidelines advocate routine screening for depression. Yet this recommendation is unlikely to be widely adopted without trial evidence that depression care improves clinical outcomes, and efficient and reimbursable methods to provide it in typical practice settings.

Strategies to treat depression in cardiac populations are of great interest given their potential, in theory, to reduce morbidity. Unfortunately, most interventions evaluated in clinical trials, including some conducted in HF patients, had little or no impact at reducing mood symptoms. Possible explanations include: (1) dependence solely on single antidepressant agents that, in general, are often ineffective, untolerated, or discontinued by patients; (2) reliance on psychological counseling in medically-ill populations who may be either unwilling or unable to adhere to successive face-to-face encounters with a therapist; (3) inadequate consideration of patients’ preferences for type and location of treatment; (4) insufficient attention to adherence with recommended care; (5) perceived stigma of depression; (6) brief duration of treatment and follow-up; and (7) higher than expected spontaneous remission rates for depression.

Our NHLBI-funded “Bypassing the Blues” (BtB) Trial was the first comparative-effectiveness trial of a collaborative care strategy for treating depression following an acute cardiac event (Rollmen BL, et al. JAMA 2009). Study nurses screened post-CABG patients for depression at 7 Pittsburgh-area hospitals with the PHQ-2, and two weeks later telephoned those screen-positive to confirm the presence of at least a moderate level of mood symptoms (PHQ-9 ≥ 10). We then randomized 302 patients to either an 8-month course of telephone-delivered “stepped” collaborative care for depression (C9b-d) or to their doctors’ UC, and enrolled another 151 non-depressed post-CABG patients who served as a comparison cohort (PHQ-2 (-) and PHQ-9 <5) (Total N=453). At 8-month follow-up, intervention patients had significant improvements in HRQoL (SF-36 MCS ES: 0.30; p=0.01) and mood (Hamilton Rating Scale for Depression ES: 0.30; p=0.009) compared to UC. The 0.30 ES improvement in mood symptoms produced by the BtB intervention compared favorably to the improvements in mood symptoms reported by other trials of depression treatments among patients with CVD. However, BtB was the only depression-CVD trial that: (1) was linked to primary care; (2) required patients to obtain their own pharmacotherapy and mental health specialty (MHS) care at cost; and (3) conducted a cost-effectiveness analysis. We analyzed insurance claims data from Medicare and the two largest health insurance companies in western Pennsylvania. Estimating a $460 per patient cost for our intervention, we reported that treated patients had $449 lower mean total costs at 12-month follow-up than those randomized to UC ($18,172 vs. $18,621) at a highly favorable negative incremental cost effectiveness ratio of -$9,889 per QALY (i.e., more QALYs and at a lower cost). We are unaware of any trial that reported the effectiveness of collaborative care for depression in HF patients. Still, even if collaborative care for treating depression in HF patients were proven effective, it may still be impractical for health care delivery systems to support and patients to participate in separate treatment programs for depression and HF. Thus we are encouraged by recent evidence that indicates blended strategies for treating depression and co-morbid diabetes may produce greater improvements in mood symptoms and control of cardiovascular risk factors than programs focused solely on treating depression.

1.4 Significance: Why is it important that this research be conducted? What gaps in existing information or knowledge is this research intended to fill?

Improving chronic illness care for medically complex patients is one of the major challenges facing medicine today as patients with multiple chronic diseases account for the majority of health care costs. Indeed, 80% of HF patients have four or more chronic conditions and it would be impractical to deploy separate collaborative care programs for each. Perhaps due to the bidirectional adverse impact of depression on co-morbid medical illness, emerging evidence indicates that “blended” interventions that focus on treating both psychiatric and physical conditions produce greater improvements in mood symptoms and control of cardiovascular risk factors than programs focused solely on treating depression. Indeed, if proven effective and cost-effective, our potentially more powerful, scalable, and efficient “blended” care approach for treating HF and co-morbid depression may have profound implications for improving chronic illness care and stimulate development of “blended” interventions for other clusters of related medical conditions. HF programs are being increasingly deployed by integrated health care delivery systems and emerging “accountable care organizations” to reduce the risk of hospital readmissions. Thus if we can demonstrate that our “blended” care strategy significantly improves patient outcomes over conventional HF care, then providing effective depression care through existing HF treatment programs will be far more practical means to do so than a separate system focused solely on treating depression that could prove difficult to deploy and sustain in routine practice.

2.0 – Research and Design Methods

2.1 Does this research study involve the use or evaluation of a drug, biological, or nutritional (e.g., herbal or dietary) supplement?

No.

2.2 Will this research use or evaluate the safety and/or effectiveness of one or more devices?

No

2.2.1 Does this research study involve an evaluation of the safety and/or effectiveness of one or more devices not currently approved by the FDA for general marketing?

No

2.3 Summarize the general classification (e.g., descriptive, experimental) and methodological design (e.g., observational, cross-sectional, longitudinal, randomized, open-label single-blind, double-blind, placebo-controlled, active treatment controlled, parallel arm, cross-over arm) of the proposed research study, as applicable.

Our protocol is a randomized controlled effectiveness trial comparing: (1) collaborative care for treating both HF and depression (“blended”); (2) collaborative care for treating HF alone (enhanced usual care (eUC)); or (3) their doctors’ “usual care” for treating HF and depression (UC). We will also follow-up with a non-depressed control group.

2.3.1 Does this research study involve a placebo-controlled arm?

No.

2.4 Will any research subjects be withdrawn from known effective therapy for the purpose of participating in this research study?

No.

2.5 Will screening procedures (i.e., procedures to determine research subject eligibility) be performed specifically for the purpose of this research study?

Yes.

2.5.1 List the screening procedures that will be performed for the purpose of this research study. Do NOT include the inclusion/exclusion criteria in this section as they will be addressed in section 3; questions 3.13 and 3.14.

Each UPMC hospital has an inpatient HF Management Program that HF patients are referred to automatically upon hospital admission through an automated review of their admitting diagnoses, or following admission should the HF diagnosis be uncovered during the hospitalization. A study nurse will then explain our study, and after obtaining verbal consent, will assess the patient for the following eligibility criteria prior to hospital discharge. They include:

1. New York Heart Association (NYHA) screen to assess the severity of HF symptoms. If the patients reports class II-IV HF symptoms, then
2. the study nurses will then administer the two-item PHQ-2 depression screen, as an efficient screen for HF patients for depression. Patients answering "yes" to either item 2 will be considered PHQ-2 screen positive (PHQ-2 (+)), and eligible to continue. We will then
3. Administer the Montreal Cognitive Assessment (MoCA), to confirm that no cognitive impairment or organic mood syndrome, including those secondary to medical illness or drugs as documented in the record, use of donepezil or similar medications for treating cognitive impairment exist. And then we will establish that the patient:
4. Is able to be evaluated and treated for depression as an outpatient.
5. Is English speaking, not illiterate, or possessing any other communication barrier.
6. Has a household telephone.
7. Is not receiving active treatment for a mood or anxiety disorder from a mental health specialist.
8. Is in no unstable medical condition as indicated by history, physical, and/or laboratory findings.
9. Is not currently suicidal.
10. Has no current or history of psychotic illness according to DSM-IV criteria.
11. Has no current or history of bipolar illness according to patient self-report, past medical history, and DSM-IV criteria.
12. Has no current alcohol or other substance abuse as evidenced by chart review and the AUDIT-C questionnaire.
13. Is age >= 21 years old.

This will take up to 30 minutes. If the patient answers "no" to both items and scores <4 on the PHQ-9 indicating an absence of mood symptoms and wishes to continue, they will be randomly sampled to participate in our non-depressed comparison cohort (see Figure 1 in grant application). All PHQ-2 (+) patients will receive their doctors’ “usual” post-discharge medical care and a copy of the NIMH brochure entitled “Depression and Heart Disease” or a similar pamphlet, to destigmatize depression, increase awareness of the condition, and briefly describe treatment options. Regardless of whether the patient later agrees to enroll into our study. At 2-weeks following hospital discharge, a member of our study team will recontact the patient via telephone to confirm eligibility criteria and administer the PHQ-9. For PHQ-2 (+) patients we will

require a PHQ-9 score of 10 or higher to continue in our protocol; and for PHQ-2 (-)/PHQ-9 <5 patients, we will require a PHQ-9 <5 to continue in our non-depressed comparison cohort.

2.5.2 What steps will be taken in the event that a clinically significant, unexpected disease or condition is identified during the conduct of the screening procedures?

When suicidal ideation is uncovered during one of these routine contacts (e.g., on the HRS-D), our paperless data management system will automatically launch our IRB-approved suicidal protocol. The clinical assessor will follow the protocol and probe to determine the frequency, chronicity, and content of the ideation and then immediately page Dr. Karp and/or Dr. Herbeck Belnap, our study psychiatrist and psychologist, respectively, to discuss the patient’s situation. Afterwards, one of them or Dr. Rollman will page the patient’s PCP to inform the PCP of the situation, provide treatment advice, and arrange follow-up as clinically necessary. Per our protocol, we will follow-up with the patient 1-, 3-, 7-, and 30 days later to monitor his/her clinical status and to confirm that he/she followed-up with the recommended care plan. In addition, the initial notices sent to each patient’s PCP will advise them to evaluate their patient for suicidal ideation at each clinical encounter, and if present, to refer them for psychiatric follow-up immediately. Although Drs. Rollman, Karp, Herbeck Belnap, and the other co-investigators will not otherwise directly interact with a patient’s physician(s) to direct care, they and our care managers will assist any patient or his/her PCP by providing them with the names and phone numbers of MHSs near the patient’s home upon request and answer questions about a patient’s psychiatric condition as appropriate. While we expect to encounter few subjects with acute delirium in the course of conducting routine telephone follow-up assessments (our staff encountered 2 delirious subjects in our CABG trial and none in our pilot R34), delirium remains a marker for increased mortality and requires prompt clinical follow-up. Therefore, we will train our assessors and care managers to raise their awareness and to consider delirium when encountering a subject responding inappropriately during an interview, and to immediately inform his/her primary care physician and record these events for later analysis.

2.6 Provide a detailed description of all research activities (e.g., all drugs or devices; psychosocial interventions or measures) that will be performed for the purpose of this research study. This description of activities should be complete and of sufficient detail to permit an assessment of associated risks. At a minimum the description should include:

- **personnel (by role) performing the procedures**
- **location of procedures**
- **duration of procedures**
- **timeline of study procedures**

Our study nurses will administer our "Month 0" inpatient assessment after confirming preliminary eligibility and obtaining patients’ informed consent to enroll into our study prior to hospital discharge. Afterwards, a trained assessor who is blinded as to each patient’s baseline depression and study assignment will conduct all subsequent telephone assessments on a recorded phone line directly into our paperless data management system according to the schedule depicted in Table 3 of our grant proposal (month 0, 0.5, 3-, 6-, 12-months and then every 6 months until the conclusion of the Intervention Phase of our Trial).

Following confirmation of all protocol-eligibility criteria, our paperless data management system will randomize 625 depressed HF patients (PHQ-2 (+)/PHQ-9 >=10) to one of 3 study arms according to a pre-determined sequence stratified by study hospital developed in advance

by our study statistician. The software will be programmed to send an e-mail message to the project coordinator informing him/her of the study assignment. The project coordinator will then forward this information to the appropriate study nurse-care manager who will then inform the subject of their assignment via telephone, and also notify their PCP and/or cardiologist.

If the patient is randomized to one of our interventions (“blended” or eUC), then the care manager will telephone the subject to introduce themselves and inform them a) that he or she will be in regular contact; and b) collaborate with their PCP and/or cardiologist to help provide and monitor guideline-recommended care for depression, HF, or both depending upon study assignment under the direction of the investigators and with their doctors’ approval. However, if the patient is randomized to usual care control, then the nurse-care manager will inform the patient that we will notify their PCP and/or cardiologist about their treatment assignment, but the study will not attempt to alter in any way the care they may receive from their physicians (e.g., counseling, pharmacotherapy, or mental health specialty referral for depression or for treatments for HF). However, that we will inform their doctor only if their medical or psychiatric condition has significantly worsened during a routine blinded telephone follow-up assessment (e.g., chest pain, suicidality).

Over a 12-month time period following randomization, the nurse-care manager will call all patients randomized to an intervention arm (“blended” or eUC) on a regular basis, every other week during the acute phase of their intervention (approximately 2-3 months), and then monthly during the “continuation phase” until the end of intervention enrollment, and we expect a median of 14 calls as in our other trials. Should a patient’s symptoms (either depression or HF) worsen during the continuation phase, then the care manager will resume to more frequent calls. If a patient is not interested in the study after the first call, the patient will remain eligible for the blinded assessment calls, but he or she will not receive any more intervention calls by the nurse-care manager. We anticipate most telephone contacts between the nurse-care manager and the patient will be brief (15-20 minutes), unless special circumstances intervene (e.g., the patient expresses suicidal ideations, has questions or side effects regarding pharmacotherapy). For all intervention patients the nurse-care manager will : (1) educated the patient regarding HF and treatment goals (e.g., 20-40 min aerobics 3-5x/week); (2) encourage heart healthy lifestyle (tobacco cessation, sleep, etc.); (3) promote adherence/adjustment of HF pharmacotherapy as prescribed by the patient’s PCP/cardiologist; (4) promote outpatient self-monitoring of weight, BP, diet, and doctor visits;(5) monitor for treatment response and relapse ; and (6) suggest/facilitate PCP/cardiologist/ER referrals when appropriate. The care manager will record above data into our electronic data base and will present these at our weekly case reviews. At these meetings, Drs. Muldoon (internist), and Herbeck Belnap (study coordinator) will make recommendations regarding their pharmacotherapy for HF, promotion of healthy lifestyles (e.g., exercise, sleep, weight control, and/or referrals to PCP/Cardiologist ER) which the care manager in turn will communicate to the patient.

In addition to the above, if the patient is randomized to the "blended: intervention arm, the nurse-care manager will also discuss at the regular telephone calls: (1) psychoeducation and treatment options including pharmacotherapy and/or referral to a mental health specialist for treatment of depression; (2) discuss self-management materials (e.g., use of a workbook or the computerized cognitive behavioral therapy (CCBT) program "Beating the Blues"); (3) promote adherence/adjust antidepressant pharmacotherapy in concert with PCP; and (4) monitor for treatment response and relapse with the PHQ-9. Again, the care manager will record the above data in our data base and present these at our weekly care reviews. At these meetings, Drs. Rollman (internist), Muldoon (internist), Karp (psychiatrist), and Herbeck Belnap (psychologist and study coordinator) will make recommendations for the patient’s HF treatment as described for the “enhanced UC” patients earlier, plus they will make treatment recommendations (e.g. specific lesson plans, adjustment of pharmacotherapy, MHS referral) which the care manager in turn will communicate to the patient. After discussing the recommendation(s) with the patient

and assessing their treatment preference, the care manager will then relay the recommendation to the patient’s PCP or cardiologist for their approval via telephone, fax, or mail as appropriate that they will be free to accept, modify, or decline. The care manager will then convey the physician’s decision to the patient. However, whether the physician agrees or disagrees with the study team’s recommendation will not influence patient’s enrollment in the study.

2.7 Will follow-up procedures be performed specifically for research purposes? Follow-up procedures may include phone calls, interviews, biomedical tests or other monitoring procedures.

Yes. Trained research assessors who are blinded as to all subjects’ treatment assignment and depression status will conduct all follow-up telephone assessments on a recorded phone line directly into our paperless data management system according to the schedule depicted in Table 3 of our grant proposal (month 0.5, 3-, 6-, 12-months and then every 6 months until the conclusion of the Intervention Phase of our Trial). We anticipate each follow-up assessment will take 15-30 minutes depending upon the patient's speed in answering questions.

2.8 Does this research study involve the use of any questionnaires or survey instruments not listed in Appendix G of the IRB Reference Manual?

Yes, listed below:

Name:
HPQ
MOS Adherence Scale
Perceived Stress Scale
PHQ-9
NYHA
MoCA
Eligibility Form
PSSS
KCCQ

2.9 If subjects are also patients, will any clinical procedures that are being used for their conventional medical care also be used for research purposes?

No

2.10 Will blood samples be obtained as part of this research study?

No

2.11 What is the total duration of the subject's participation in this research study across all visits, including follow-up surveillance?

The minimum follow-up will be 12 months. However, depending on the time of the enrollment, patients enrolled at the beginning of the study will be followed up for up to 48 months maximum.

2.12 Does this research study involve any type of planned deception?

No

2.13 Does this research study involve the use of UPMC/Pitt protected health information that will be de-identified by an IRB approved "honest broker" system?

No

2.14 Will protected health information from a UPMC/Pitt HIPAA covered entity be accessed for research purposes or will research data be placed in the UPMC/Pitt medical record?

Yes.

If you answer Yes, you are required to submit this study to the Center for Assistance in Research using e-Record (CARe). Per UPMC Policy HS-RS0005, all research projects that access or involve UPMC electronic protected health information (e-PHI) must be submitted to CARe, with the exception of clinical trials that are contracted through the UPMC Office of Sponsored Programs and Research Support (OSPARS). Complete the online submission form at http://www.eresearch.pitt.edu/request.aspx. After the study is submitted in OSIRIS, a CARe representative will conduct a review. You will be notified once your CARe review is complete or if anything further is needed. For additional information, please see http://www.eresearch.pitt.edu/.

Describe the medical record information that will be collected from the UPMC/Pitt HIPAA covered entity and/or the research-derived information that will be placed in the medical records.

We inform the clinical team taking care of the patent in hospital of the results of their PHQ-2/PHQ-9 depression screen in a brief note placed on the patient's chart that will encourage the team to follow-up with the patient on this finding, if necessary. Our study nurses will also abstract portions of the medical record on all patients who meet inpatient protocol-eligibility to continue in our trial and consent to enroll prior to hospital discharge (e.g., medications, medical diagnoses, lab results). However, we will only collect information on tests that have been ordered as part of routine care and will never request any tests be ordered for research purposes. Our blinded telephone assessors will routinely ask study patients about any hospitalizations, cardiac events, or mental health visits they may have experienced since their last assessment at UPMC and non-UPMC facilities. As in our R34 pilot study (IRB 0701041), we will request the UPMC e-Record team to send us an automatically-generated hospital discharge summary anytime a study patient is discharged from one of the 13 UPMC acute care hospitals located across western Pennsylvania over the duration of their study participation. Our physician-led “Key Events Classification Committee” will then classify the event cause using a detailed protocol we developed. At the end of the Intervention Phase of our Trial, we will obtain claims data from UPMC Health plan. Specifically, we will request information on the type of service, service dates, amount paid to the provider, beneficiary copayment, and primary and secondary ICD-9 and CPT4 codes for each episode of care over the course of their enrollment so we may conduct our Project’s economic analyses (C14c).

2.14.1 Will protected health information from a non-UPMC/Pitt HIPAA covered entity be obtained for research purposes or will research data be placed in the non-UPMC/Pitt medical record?

Yes

If Yes, describe how the HIPAA requirements will be met:

As described in 2.14, our blinded telephone assessors will routinely ask study patients about any hospitalizations, cardiac events, or mental health visits they may have experienced since their last assessment at UPMC and non-UPMC facilities. Whenever a death, hospitalization, or other cardiac or potentially life-threatening event is discovered, we will obtain the relevant medical records also from non-UPMC facilities, so that our physician-led “Key Events Classification Committee” can classify the event cause using a detailed protocol we developed.

At the end of the Intervention Phase of our Trial, we will obtain claims data from Medicare (via the Research Data Assistance Center (ResDAC); http://www.resdac.org/AboutUs/Index.asp), and Highmark Blue Cross/Blue Shield. Specifically, we will request from subjects’ insurers information on the type of service, service dates, amount paid to the provider, beneficiary copayment, and primary and secondary ICD-9 and CPT4 codes for each episode of care over the course of their enrollment so we may conduct our Project’s economic analyses (C14c).

I, Bruce Rollman, certify that any member of my research team accessing, reviewing and/or recording information from medical records have completed HIPAA Researchers Privacy Requirements (Formerly RPF Module 6) training. The HIPAA certificates must be available for review if audited but do not need to be uploaded into this OSIRIS application.

Yes

2.14.2 Are you requesting a waiver of the requirement to obtain written HIPAA authorization for the collection of the PHI from a UPMC/Pitt covered entity? Note that the University of Pittsburgh IRB cannot grant a HIPAA waiver for entities outside of UPMC/Pitt.

No

2.15 Does this research study involve the long-term storage (banking) of biological specimens?

No

2.16 Will research participants be asked to provide information about their family members or acquaintances?

Yes

2.16.1 Describe what information about the third party will be obtained from the participant:

We will ask for secondary contact information (spouse, adult child, friend) so that we may contact patients in the event we are unable to reach the patient at a later date (e.g., 2-week follow-up). We will not ask for any other information about any third party.

2.16.2 If the information about the third party is of a private nature, can the identity of the third party be readily ascertained or associated with this information?

Yes.

Describe the private information that will be collected and recorded about the third party:

Name, phone number, relationship to study patient.

2.17 What are the main outcome variables that will be evaluated in this study?

HRQoL is increasingly recognized as an important outcome, particularly for disorders causing disabling daily symptoms. We selected the generic and widely used SF-36 Mental Component Summary (MCS) as our primary outcome measure of HRQoL as: (1) we and others have demonstrated its sensitivity to changes in mood; (2) it has been validated for use in patients with HF; and (3) it allows us to measure the impact of our “blended” intervention on two conditions simultaneously. Since a more disease-specific quality of life measure can sometimes provide a more sensitive indicator of program benefit, we also selected the Kansas City Cardiomyopathy Questionnaire (KCCQ) and the version of the Hamilton Rating Scale for Depression (HRS-D).

2.18 Describe the statistical approaches that will be used to analyze the study data.

We will evaluate the comparative effectiveness of our interventions to address our co-primary hypotheses on HRQoL and a broad variety of other outcomes of interest (e.g., HRS-D) using ITT mixed-effects repeated measures models with time (random) gender, treatment, and interaction terms. We will also evaluate time to first hospitalization, death, and composite major adverse cardiac event (all-cause death, MI, stroke, and rehospitalization for cardiac cause) using Kaplan-Meier survival analyses with non-directional 2-tailed log-rank tests to evaluate differences by study arm. We will apply similar techniques to describe and compare subjects in our non-depressed cohort to depressed randomized study subjects. We will first examine our data by study arm in univariate fashion using cross tabulations, histograms, and box plots applying standard statistical tests to assess covariate balance by randomization status and recruitment site, and adjusting for baseline differences in our multivariable models where appropriate. To handle missing data, we will first investigate the missingness process and investigate the reasons for dropouts, and use a likelihood-based or multiple imputation-based procedure if "missing at random" (MAR) is confirmed or consider selection models with a logistic dropout process or other model as found appropriate if the missingness (dropout) is nonignorable (missing not at random (MNAR)).

2.19 Will this research be conducted in a foreign country or at a site (e.g., Navajo Nation) where the cultural background of the subject population differs substantially from that of Pittsburgh and its surrounding communities?

No

2.21 Will this research study be conducted within a nursing home located in Pennsylvania?

No.

3.0 – Human Subjects

3.1 What is the age range of the subject population?
Age 21 and above

3.2 What is their gender?
Both males and females.

3.3 Will any racial or ethnic subgroups be explicitly excluded from participation
No

3.4 For studies conducted in the U.S., do you expect that all subjects will be able to comprehend English?
Yes.

3.5 Participation of Children: Will children less than 18 years of age be studied?
No.

If No, provide a justification for excluding children.
Based on a review of the age distribution of patients admitted for HF at our study hospitals (youngest ~mid-30’s) and our R34 HF pilot data, we do not expect to encounter any child or adolescent patients (age < 21) on the adult inpatients wards where we plan to conduct our enrollment procedures. Furthermore, given the outlier status these participants would present, we will exclude children and adolescents from our protocol.

3.6 Does this research study involve prisoners, or is it anticipated that the research study may involve prisoners?
No.

3.7 Will pregnant women be knowingly and purposely included in this research study?
No.

3.8 Does this research study involve neonates?
No.

3.9 Fetal Tissues: Does this research involve the use of fetal tissues or organs?
No.

3.10 What is the total number of subjects to be studied at this site, including subjects to be screened for eligibility? Note: The number below is calculated by summing the data entered in question 3.11. Any additions or changes to the values entered in 3.11 will be reflected in 3.10.

4500

3.11 Identify each of the disease or condition specific subgroups (include healthy volunteers, if applicable) that will be studied.

1) how many subjects will undergo research related procedures at this site; and

2) if applicable, how many subjects will be required to undergo screening procedures (e.g., blood work, EKG, x-rays, etc.) to establish eligibility. Do Not include subjects who will undergo preliminary telephone screening.

Subgroup	Number to undergo research procedures	Number to undergo screening procedures	
Control	125	750	125<br>750
eUC	250	1500	250<br>1500
UC	125	750	125<br>750
Blended	250	1500	250<br>1500

3.12 Provide a statistical justification for the total number of subjects to be enrolled into this research study at the multicenter sites or this site.

If applicable, refer to the statistical section in the clinical protocol or grant. Specify the page numbers in the textbox.See C13a-f in our grant application.

3.13 Inclusion Criteria: List the specific criteria for inclusion of potential subjects.

1. Systolic heart failure (documented ejection fraction ≤ 40%).
2. HF symptoms meeting criteria for New York Heart Association (NYHA) classes II, III or IV.
3. Inpatient PHQ-2 screen-positive for depression; or PHQ-2 screen negative for depression and PHQ-9 <5 if non-depressed control.
4. PHQ-9 ≥ 10 when reassessed two-weeks following hospital discharge, or PHQ-9 <5 if non-depressed control.
5. No cognitive impairment (as documented in the record, use of donepezil or similar medications for treating cognitive impairment, or the MoCA).
6. Able to be evaluated and treated for depression as an outpatient.
7. English speaking, not illiterate, or possessing any other communication barrier.
8. Have a household telephone.

3.14 Exclusion Criteria: List the specific criteria for exclusion of potential subjects from participation.

1. Receiving active treatment for a mood or anxiety disorder from a mental health specialist.
2. Unstable medical condition as indicated by history, physical, and/or laboratory findings.
3. Presence of non-cardiovascular conditions likely to be fatal within 12 months (e.g., cancer).
4. Organic mood syndromes, including those secondary to medical illness or drugs.
5. Active suicidal ideation.
6. Current or history of psychotic illness according to DSM-IV criteria.
7. Current or history of bipolar illness according to patient self-report, past medical history, and DSM-IV criteria.
8. Current alcohol or other substance abuse as evidenced by chart review and the AUDIT-C questionnaire.
9. Age $\leq$ 21 years.

3.15 Will HIV serostatus be evaluated specifically for the purpose of participation in this research study?

No.

<u>4.0 – Subject Recruitment and Informed Consent Procedures</u>

4.1 Select all recruitment methods to be used to identify potential subjects:

Waiver of informed consent to review medical records ONLY for the identification of potential subjects.

Advertisements.

4.2 Provide a detailed description of your recruitment methods, including identifying and initiating contact with participants:

As described earlier in this IRB application, each UPMC hospital has an inpatient HF Management Program that HF patients are referred to automatically upon hospital admission through an automated review of their admitting diagnoses, or following admission should the HF diagnosis be uncovered during the hospitalization. Our nurses will be notified by the house staff of these patients, upon which the study nurses will confirm the patient's ejection fraction. If it is >40%, then the nurses will ask the house staff to get that patient's permission to approach. If the patient agrees to be approached our study nurse will then explain our study, and after obtaining verbal consent, will assess the patient for the described eligibility criteria prior to hospital discharge. If the patient is not interested in our study, we will only collect his or her data as required by our funding organization (age, EF, race, gender).

4.6 Are you requesting a waiver to document informed consent for any or all participants, for any or all procedures? (e.g., a verbal or computerized consent script will be used, but the subjects will not be required to sign a written informed consent document, such as with phone screening. This is not a waiver to obtain consent.

Yes

4.6.1 Identify the specific research procedures and/or the specific subject populations for which you are requesting a waiver of the requirement to obtain a signed consent form. If not all, identify the specific procedures and/or subject populations for which you are requesting a waiver:

We request a waiver of signed consent for our study nurses to determine whether they may later be eligible to provide written informed consent to participate in our clinical trial. Our nurses will briefly describe the study, and if they patient is interested, they will establish eligibility (e.g., having a telephone, no history of severe mental illness), and administer the PHQ-2, NYHA questions, to establish if the patient indeed meets criteria for our study. We will also ask the patient their sex and race in order to be able to report these data to our funding agency. Finally we will administer the Montreal Cognitive Assessment to establish that eligible patients will be able to understand the study consent document, Data collected during this screening will be de-identified, assigned a unique subject ID number, and stored electronically on a password protected laptop.

4.6.2 Indicate which of the following regulatory criteria is applicable to your request for a waiver of the requirement to obtain a signed consent form.

45 CFR 46.117(c)(2)

45 CFR 46.117(c)(1) That the only record linking the subject and the research would be the consent document and the principal risk would be potential harm resulting from a breach of confidentiality. Each subject will be asked whether the subject wants documentation linking the subject with the research, and the subject's wishes will govern; or

45 CFR 46.117(c)(2) That the research presents no more than minimal risk of harm to subjects and involves no procedures for which written consent is normally required outside of the research context.

4.6.2.1 Address why the specific research procedures for which you are requesting a waiver of the requirement to obtain a signed consent form present no more than minimal risk of harm to the research subjects:

There are no physical or psychological risks associated with this research activity, and patients can decline to participate in our screening procedure and later in the trial at any time. The questions we propose to ask them are similar to those questions asked upon scheduling them for a medical or psychiatric clinic visit.

4.6.2.2 Justify why the research listed in 4.6.1 involves no procedures for which written informed consent is normally required outside of the research context:

HF patients who are suspected to have developed symptoms of depression are routinely screened with the PHQ-2 and PHQ-9 as recommended by an American Heart Association Science Advisory. Therefore, our screening questions are not additional burden to the patient, and should be administered in routine care.

4.6.3 Address the procedures that will be used and the information that will be provided (i.e., script) in obtaining and documenting the subjects' verbal informed consent for study participation:

The recruiter will briefly explain the purpose of the research study and ask the subject if they are interested. The answer will be documented in our paperless data entry system which will

also guide the recruiter through the remainder of the screening process. (Screening Script attached in IRB form).

4.7 Are you requesting a waiver to obtain informed consent or an alteration of the informed consent process for any of the following?

Yes.

4.7.1 If Yes, select the reason(s) for your request:

Medical record review for ONLY the identification of potential subjects

General Requirements: The Federal Policy [45 CFR 46.116 (d)] specifies in order for a waiver of consent to be approved, the request must meet four criteria. For each request, you will be asked to provide a justification addressing how each of these criterion is met.

Medical record review for the identification of potential subjects:

The research involves no more than minimal risk to the subjects; [45 CFR 46.116 (d)(1)]	The review of medical record information to determine eligibility poses no more than minimal risk to subjects since no changes will be made to patient care, and only those individuals who have clinical access will review records to determine eligibilty. The research data (ejection fraction) will be entered in a password protected laptop into a password protected database that will be housed on a secure server of our Data Center.Records will be reviewed for eligibility only, and only by members of the research team who also has clinical access to the records, so patient privacy will not be violated.
The waiver or alteration will not adversely affect the rights and welfare of the subjects;[45 CFR 46.116 (d)(2)]	Records will be reviewed for eligibility only, and only by members of the research team who also has clinical access to the records, so patient privacy will not be violated.
The research could not practicably be carried out without the waiver or alteration;[45 CFR 46.116 (d)(3)]	The patients whose records will be viewed for eligibility are ill and hospitalized; we do not want to burden them by obtaining signed consent before it is clear that they are eligible for the first phase of screening.
Whenever appropriate, the subjects will be provided with additional pertinent information after participation; [45 CFR 46.116 (d)(4)]	Eligible patients will be approached for consent to the larger study. Our study nurses will only confirm the percentage of their ejection fraction. Given the limited use of this Waiver, it is unlikely that pertinent information will be obtained.

Review of identifiable medical records: [Note: A waiver of HIPAA Authorization must be requested (2.14.2)]

The research involves no more than minimal risk to the subjects;[45 CFR 46.116 (d)(1)]

The waiver or alteration will not adversely affect the rights and welfare of the subjects;[45 CFR 46.116 (d)(2)]

The research could not practicably be carried out without the waiver or alteration;[45 CFR 46.116 (d)(3)]

Whenever appropriate, the subjects will be provided with additional pertinent information after participation.[45 CFR 46.116 (d)(4)]

Parental Permission and/or Child Assent

The research involves no more than minimal risk to the subjects;[45 CFR 46.116 (d)(1)]

The waiver or alteration will not adversely affect the rights and welfare of the subjects;[45 CFR 46.116 (d)(2)]

The research could not practicably be carried out without the waiver or alteration;[45 CFR 46.116 (d)(3)]

Whenever appropriate, the subjects will be provided with additional pertinent information after participation.[45 CFR 46.116 (d)(4)]

Alteration of informed consent process

The research involves no more than minimal risk to the subjects;[45 CFR 46.116 (d)(1)]

The waiver or alteration will not adversely affect the rights and welfare of the subjects;[45 CFR 46.116 (d)(2)]

The research could not practicably be carried out without the waiver or alteration;[45 CFR 46.116 (d)(3)]

4.8 Are you requesting an exception to the requirement to obtain informed consent for research involving the evaluation of an “emergency’ procedure? Note: This exception allows research on life-threatening conditions for which available treatments are unproven or unsatisfactory and where it is not possible to obtain informed consent.

No

4.9 Upload all written informed consent documents.

Draft Consent Forms for editing (attached in IRB form):

Name
Consent Form - Depressed
Consent Form - Non-depressed

Approved Consent Form(s):

Name
Consent Form - Depressed
Consent Form - Non-depressed

4.10 Will all potential adult subjects be capable of providing direct consent for study participation?

Yes.

4.11 At what point will you obtain the informed consent of potential research subjects or their authorized representative?

After performing certain of the screening procedures, but prior to performing any of the research interventions/interactions.

4.11.1 Address why you feel that it is acceptable to defer obtaining written informed consent until after the screening procedures have been performed.

As seen in our previous R34 cohort study where we screened 857 hospitalized HF patients, only 401 (47%) met primiinary eligibility criters of NYHA class and PHQ-2 positive. Therefore, it would be an unnecessary burden for the rather sick patients to undergo the entire consent process while the majority will not be protocol eligible. We therefore request to only spend 10-15 minutes with the patient for the screening, and then spend the additional 30-45 minutes required to administer the MoCA, explain the study in detail, and obtain the written consent only with those patients who are eligible. This would also streamline our study's recruitment process, and help us meet our recruitment milestones.

4.11.2 Taking into account the nature of the study and subject population, indicate how the research team will ensure that subjects have sufficient time to decide whether to participate in this study. In addition, describe the steps that will be taken to minimize the possibility of coercion or undue influence.

If the patient meets our eligibility criteria, then our study nurse will give the patient the consent document, and offer to return after the patient had time to read the document. Afterwards, the patient will have time to ask the study nurse any questions that may occur when he/she returns to the patient's room. Patients may still request additional time to discuss their participation with family members or friends, and if so the study nurse will come back on a later day to review the consent document. We will train all study nurses in the explanation of the study, and in obtaining consent in a non-coercive manner. Furthermore, we will conduct spot-checks to ensure that no undue influence is exercised on potential study patients.

4.12 Describe the process that you will employ to ensure the subjects are fully informed about this research study. This description must include the following elements:

- **who from the research team will be involved in the consent process (both the discussion and documentation);**
- **person who will provide consent or permission;**
- **information communicated; and**
- **any waiting period between informing the prospective participant about the study and obtaining consent**

Each UPMC hospital has an inpatient HF Management Program that HF patients are referred to automatically upon hospital admission through an automated review of their admitting diagnoses, or following admission should the HF diagnosis be uncovered during the hospitalization. Our study cardiologist, Dr. Hunter Champion, will encourage HF Management Program staff and cardiology consult teams to obtain patients’ whose ejection fraction is =< 40% to provide verbal HIPAA agreement permitting our nurse-recruiters to confirm their ejection fraction meets study criteria, and if so to approach and explain our study and obtain their patients’ consent to undergo our screening procedures. Our study nurses will then screen the patients for eligibility as described in 4.2. If the patient meets eligibility criteria for our study, and remains interested, the study nurse will then explain the study in detail as outlined in the

consent document, answer any questions the patient may have, and give the patient time to consider participation or discuss it with his.her family. If the patient agrees to participate, then they will be asked to sign the informed consent, and the study nurse will document the process. At 2-weeks after hospital discharge a research assessor will call the patient, and establish if the patient is still eligible. At this point the patient can also decide to continue to participate in our study. Only if the patient is still eligible and confirms his continued interest, will he/she be randomized to our study.

4.13 Are you requesting an exception to either IRB policy related to the informed consent process? For studies involving a drug, device or surgical procedures, a listed physician investigator is required to obtain the written informed consent unless an exception to this policy has been approved by the IRB For all other studies, a listed investigator is required to obtain consent (Note: In order to request an exception to this policy, the study must be minimal risk).

Yes

If Yes, provide a justification and describe the qualifications of the individual who will obtain consent:

We request an exemption to IRB Policy 4.13 because the study involves minimal risk and the study doctors will never prescribe any drugs, devices, or surgical procedures. Requiring a study physician to obtain consult would also make the trial logistically impractical to conduct as we plan to hire study nurses who will enroll study patients from 4-6 UPMC-affiliated hospitals daily during routine weekday business hours.

4.14 Will you inform research subjects about the outcome of this research study following its completion?

Yes.

If Yes, describe the process to inform subjects of the results:

We will telephone the patient to confirm vital status and mailing address after our trial's 'main outcomes' paper is published, and if the patient is alive mail them a copy of our paper.

<u>5.0 – Potential Risks and Benefits of Study Participation</u>

5.1 Describe potential risks (physical, psychological, social, legal, economic or other) associated with screening procedures, research interventions/interactions, and follow-up/monitoring procedures performed specifically for this study:

Research Activity:	Intervention
Common Risks:	Patients may experience discomfort discussing disturbing symptoms and/or psychosocial concerns.
Infrequent Risks:	Breaches in confidentiality involving medical information could impact future insurability, employability, or reproduction plans, or have a negative impact on family relationships, and/or result in stigmatization.
Other Risks:	Disclosure of suicidal behavior and medical problems

Research Activity:	Screening and assessment
Common Risks:	The discussion of personal problems during screening could be stressful, uncomfortable, or embarrassing to some people. They may be falsely labeled as having a mood disorder.
Infrequent Risks:	Breaches in confidentiality involving medical information could impact future insurability, employability, or reproduction plans, or have a negative impact on family relationships, and/or result in stigmatization.
Other Risks:	No Value Entered

5.1.1 Describe the steps that will be taken to prevent or to minimize the severity of the potential risks:

The risks to study patients are that they may experience discomfort discussing disturbing symptoms and/or psychosocial concerns, and that they may be falsely labeled as having a mood disorder and offered treatment for it. Furthermore, stigmatization surrounding mental illness can make exploration of emotional issues uncomfortable, particularly if the patient has not established a secure relationship with his/her cardiologist and PCP. Still, given the 85% specificity of the PHQ-2/PHQ-9 screening procedure for major depression among patients with CVD, we believe the great majority of our findings will be correct on those patients so identified. The few subjects who are inappropriately diagnosed as having depression may, in fact, have a partly remitted episode or a subsyndromal disorder.

Since these conditions are associated with significant impairment in quality of life, patients so “overdiagnosed” may still benefit from any increased attention provided to them by their PCP or from our interventions. With regard to experiencing discomfort when discussing disturbing psychosocial concerns, our assessor and care manager will advise subjects who are unduly distressed to contact their PCP, or Drs. Rollman, Karp, or Herbeck Belnap to discuss these concerns. Patients may voice medical problems (e.g., chest pain) or suicidal ideation during their telephone encounters with study staff. In case of physical problems, our staff will advise the patient to call their physician right away to first clarify the severity of their medical problems. If a patient voices suicidal ideation, our electronic suicide risk management protocol (Herbeck Belnap et al. 2011) that we have used in our previous studies will be automatically triggered, and the staff will follow its guidelines. Furthermore, we will again establish an on-call system of study psychiatrists who will be available 24/5 for the staff for triage.

All research staff and study investigators will complete the University of Pittsburgh’s on-line training for the ethical conduct of clinical research (http://www.health.pitt.edu/rpf/), and we will obtain approval from our University IRB which covers all UPMC clinical facilities prior to the commencement of study recruitment. Detailed descriptions of patient recruitment and informed consent are provided in C3-C4.No patient or physician will be identified in any description or publication of this research.

Confidentiality will be maintained by assigning each study patient a computer-generated identifying number for use in our data files. All data obtained will be kept in locked file cabinets and on password-protected computers with access provided only to designated study personnel to ensure strict confidentiality. Select members of our research team will have electronic access to specific folders that contain de-identified patient data (e.g., database manager and statistician). Identified data will be stored in a separate table with only the database manager, assessor, and care manager having access in accordance with their roles on the project (e.g., to send-out participant payments). No personal information concerning study subjects will be released without their written consent, with the exception should one of our study staff detect active suicidality and/or acute clinical decompensation which will be released only to the

subject’s personal physician(s). We will store paper consent forms, discharge summaries, and other primary data sources in locked files at the Center for Research on Health Care where the investigators’ offices and our Data Center are located with access provided only to designated research personnel. Each study member who is provided with access to our paperless data management system (C16) will be issued a unique, permanent, user identifier (ID), and private password. Both will be needed to confirm one’s identity whenever retrieving or entering data. We will set-up different user profiles that will allow access to relevant sections of the database by job description. Sharing of passwords is strictly forbidden and leaving unattended “open” records strongly discouraged by the use of reminder messages displayed at the time of logging-in to the system and timed automatic log-outs from the system. User accounts for study staff who leave the employment of our research team will be terminated following their last day of work. Routine patient monitoring and quality control reports will only identify patients by study number and the patient’s first name as we did on the weekly monitoring reports generated for all intervention patients enrolled into our CABG study.

To further promote the security and integrity of our data, our paperless data management system will operate on a dedicated computer server in our Data Center behind the UPMC network “firewall” to protect against viruses, evesdropping, tampering, and unauthorized usage of the system. Our Data Center has expertise in developing HIPAA-compliant software applications and we will work with them to ensure our system is in compliance with all regulations.

5.2 What steps will be taken in the event that a clinically significant, unexpected disease or condition is identified during the conduct of the study?

When suicidal ideation is uncovered during one of these routine contacts (e.g., on the HRS-D), our paperless data management system will automatically launch our IRB-approved suicidal protocol. The clinical assessor will follow the protocol and probe to determine the frequency, chronicity, and content of the ideation and then immediately page Dr. Karp and/or Dr. Herbeck Belnap, our study psychiatrist and psychologist, respectively, to discuss the patient’s situation. Afterwards, one of them or Dr. Rollman will page the patient’s PCP to inform the PCP of the situation, provide treatment advice, and arrange follow-up as clinically necessary. Per our protocol, we will follow-up with the patient 1-, 3-, 7-, and 30 days later to monitor his/her clinical status and to confirm that he/she followed-up with the recommended care plan. In addition, the initial notices sent to each patient’s PCP will advise them to evaluate their patient for suicidal ideation at each clinical encounter, and if present, to refer them for psychiatric follow-up immediately. Although Drs. Rollman, Karp, Herbeck Belnap, and the other co-investigators will not otherwise directly interact with a patient’s physician(s) to direct care, they and our care managers will assist any patient or his/her PCP by providing them with the names and phone numbers of MHSs near the patient’s home upon request and answer questions about a patient’s psychiatric condition as appropriate. While we expect to encounter few subjects with acute delirium in the course of conducting routine telephone follow-up assessments (our staff ncountered 2 delirious subjects in our CABG trial and none in our pilot R34), delirium remains a marker for increased mortality and requires prompt clinical follow-up. Therefore, we will train our assessors and care managers to raise their awareness and to consider delirium when encountering a subject responding inappropriately during an interview, and to immediately inform his/her primary care physician and record these events for later analysis.

5.3 All the risk questions (screening, intervention/interaction, follow-up) have been merged into one question (5.1).

5.4 Do any of the research procedures pose a physical or clinically significant psychological risk to women who are or may be pregnant or to a fetus?

No.

5.5 Do any of the research procedures pose a potential risk of causing genetic mutations that could lead to birth defects?

No.

5.6 Are there any alternative procedures or courses of treatment which may be of benefit to the subject if they choose not to participate in this study?

No.

5.7 Describe the specific endpoints (e.g., adverse reactions/events, failure to demonstrate effectiveness, disease progression) or other circumstances (e.g., subject's failure to follow study procedures) that will result in discontinuing a subject’s participation?

When psychiatric, medical, or neurologic complications may be present, the recruiter will ask Dr. Rollman or another study physician to review the patient’s clinical record. We will reclassify any patient as “protocol-ineligible” if he/she is found in retrospect to have had alcohol dependence, an organic mood disorder, bipolar disorder, psychosis, or another condition at baseline that would be in violation of our protocol-eligibility criteria if known.

5.8 Will any individuals other than the investigators/research staff involved in the conduct of this research study and authorized representatives of the University Research Conduct and Compliance Office (RCCO) be permitted access to research data/documents (including medical record information) associated with the conduct of this research study?

No

5.9 Has or will a Federal Certificate of Confidentiality be obtained for this research study?

No

5.10 Question has been moved to 5.17

5.11 Question has been moved to 5.16

5.12 Does participation in this research study offer the potential for direct benefit to the research subjects?

Yes.

Describe the direct benefit that subjects may receive as a result of study participation. Indicate if all, or only certain, of the subjects may derive this potential benefit. Describe the benefit:

The potential benefit to subjects participating in the proposed study is that treatment can be received for an episode of depression previously unrecognized and untreated that could potentially affect the course of their HF. We will inform the patient and his/her treating

physician(s) of the finding of a screen positive PHQ-2, or elevated mood symptoms on the PHQ-9/HRS-D administered at baseline or over the course of follow-up (Table 3 of grant proposal). Patients enrolled in our trial's two active intervention arms ("blended" and eUC) will also have the opportunity to receive closer follow-up and care of their HF and/or mood disorder. In a more general sense, the development of widely generalizable strategies to recognize and treat depression in HF patients could significantly improve clinical care for this population that experiences higher rates of morbidity and mortality compared to the general population of HF patients. On balance, we believe the potential benefits of the increased knowledge gained from the study outweigh the potential risks posed given the close monitoring of all patients, and exposure of intervention patients to our collaborative care program for depression. Moreover, our treatment protocols will not involve any investigational new drugs or devices.

5.13 Describe the data and safety monitoring plan associated with this study. If the research study involves multiple sites, the plan must address both a local and central review process.

Dr. Rollman as study PI will be responsible for overseeing all aspects of our data and safety monitoring plans as described earlier. All episodes of suicidality uncovered by any member of our research team will be brought to the immediate attention of Drs. Karp and/or Herbeck Belnap, our study mental health specialists (D4d). As in our Bypassing the Blues Trial, we will have a Patient Safety Coordinator and physician-led Key Events Classification Committee to adjudicate the cause for each serious event identified by our blinded telephone assessors, and forward this information to the attention our DSMB, NHLBI Program Officer, and local IRB in a timely manner. Our paperless data management system should allow us to process these events faster than if we "carved-out" this function to an outside organization.

We will also form an external data safety monitoring board (DSMB) soon after our application is funded. It will have members with relevant experience in cardiology, psychiatry, and statistics who are approved by the Institute. The DSMB will review our study protocol before implementation, and then interim reports on recruitment, adverse events, and data relevant to risk-benefit and other aspects of the progress of our trial every 6 months or as the Board requests (e.g., retention and follow-up assessment rates). As with our CABG trial, we anticipate most contacts with our DSMB will be via conference call and e-mail as necessary between meetings. Our paperless data management system is capable of generating real-time reports by study ID number on all subjects whose follow-up blinded HRS-D score increases by more than 25% from their baseline score at any time. Our project coordinator will run these reports on at least an every other week basis and examine these subjects' study records. If indicated, he or she will bring them to the attention of Drs. Rollman, Karp, or Herbeck Belnap for discussion.

As per our University's IRB guidelines, if any adverse events are identified we will report this information to our IRB, the funding Institute, and forward a report to our DSMB. In this report we will include a summary of adverse event and how we handled each situation. Our paperless data management system will also allow us to track and process each key events (or serious adverse event) through the process of collection, physician adjudication, and presentation to our DSMB faster than if these processes were "carved-out" to an outside organization. In the course of conducting routine telephone assessments or care contacts our blinded assessors and care managers may also encounter HF patients in the process of clinical decompensation (e.g., patient self-report of severe shortness of breath, chest pressure, etc.).

As with our CABG and HF studies, in these events our study staff will advise these patients to telephone their PCP and/or cardiologists ASAP for further advice, present to an emergency room, or call "911" for immediate medical attention given the seriousness and instability of patients with HF. As referral to the local ER may bias our findings towards an increase in the rate or number of hospitalizations in intervention patients, we will record these events to adjust for a confounding of our findings later.

5.14 What precautions will be used to ensure subject privacy is respected? (e.g. the research intervention will be conducted in a private room; the collection of sensitive information about subjects is limited to the amount necessary to achieve the aims of the research, so that no unneeded sensitive information is being collected, drapes or other barriers will be used for subjects who are required to disrobe).

No personal information concerning study subjects will be released without their written consent, with the exception of active suicidality which will be released only to the subject's personal physician(s), and no patient will be identified in any description or publication of this research. All study patients will be assigned unique study identifiers that will appear on all data collection instruments, audiofiles, documents, and other reports used for statistical analyses and manuscript preparation. We will review study procedures to protect the privacy of the subjects and the confidentiality of their data. The study coordinator will perform random checks on our study team's adherence to confidentiality to ensure that all data obtained is kept in locked file cabinets and on password protected computers.

5.15 What precautions will be used to maintain the confidentiality of identifiable information? (e.g., paper-based records will be kept in a secure location and only be accessible to personnel involved in the study, computer-based files will only be made available to personnel involved in the study through the use of access privileges and passwords, prior to access to any study-related information, personnel will be required to sign statements agreeing to protect the security and confidentiality of identifiable information, whenever feasible, identifiers will be removed from study-related information, precautions are in place to ensure the data is secure by using passwords and encryption, because the research involves web-based surveys, audio and/or video recordings of subjects will be transcribed and then destroyed to eliminate audible identification of subjects).

Our Center's Data Center will set-up different user profiles that allow staff access to relevant sections of our study database by job description and create audit trails to monitor use. Only select members of our research team will have electronic access to specific folders that contain de-identified patient data (e.g., project coordinator, database manager, and statistician). Identified data will be stored in a separate table with only the database manager and project coordinator having access. We will store paper consent forms, and other primary data sources in locked files at the Center for Research on Health Care where the investigators' offices and our Data Center are located with access provided only to designated research personnel to ensure strict confidentiality. No personal information concerning study subjects will be released without their written consent, with the exception of active suicidality which will be released only to the subject's personal physician(s), and no patient will be identified in any description or publication of this research. All study patients will be assigned unique study identifiers that will appear on all data collection instruments, audiofiles, documents, and other reports used for statistical analyses and manuscript preparation.

5.16 If the subject withdraws from the study, describe what, if anything, will happen to the subject's research data or biological specimens.

Any identifiable research or medical information recorded for, or resulting from, the participation in this research study prior to the date that a patient formally withdraws their consent may continue to be used and disclosed by the investigators for the purposes described above.

5.17 Following the required data retention period, describe the procedures utilized to protect subject confidentiality. (e.g., destruction of research records; removal of identifiers; destruction of linkage code information; secured long-term retention).

We will shred consent forms and other primary data collection instruments by 7 years after closing of the study in accordance with NIH guidelines. At that time we will also delete or destroy all other subject identifiers we may have collected and stored electronically.

6.0 – Costs and Payments

6.1 Will research subjects or their insurance providers be charged for any of the procedures (e.g., screening procedures, research procedures, follow-up procedures) performed for the purpose of this research study?

No

6.2 Will subjects be compensated in any way for their participation in this research study?

Yes.

6.2.1 Describe the amount of payment or other remuneration offered for complete participation in this research study.

Patients enrolled into the study will receive $15 for their time and effort for completing our telephone Baseline Assessment (2-weeks). Afterwards, they will receive $15 for each Follow-Up Assessment they complete (3-, 6-, and 12-months; $60 total).

6.2.2 Describe the amount and term of payment or other remuneration that will be provided for partial completion of this research study.

Patients will only be paid for assessments that they complete.

7.0 – Qualifications of Investigators and Sources of Research Study

7.1 Summarize the qualifications and expertise of the principal investigator and listed co-investigators to perform the procedures outlined in this research study.

Bruce L. Rollman, MD, MPH, Principal Investigator. Dr. Rollman is Professor of Medicine, Psychiatry, and Clinical and Translational Science, Center for Research on Health Care, Division of General Internal Medicine, University of Pittsburgh School of Medicine. His research focuses on interventions to improve the quality of care for depression and anxiety disorders in non-psychiatric settings. He has been PI on 4 R01 effectiveness trials including the first to examine the impact of collaborative care treatment for depression following CABG surgery; two NIMH-funded trials that also utilized a collaborative care treatment model; and an AHRQ-funded trial to disseminate the AHCPR’s Depression Guideline Panel’s recommendations to PCPs via an electronic medical records system. He is currently the PI on a NIMH-funded R01 that is examining the effectiveness of online treatment in primary care. Dr. Rollman also led the Clinical Model Technical Assistance Team for a Robert Wood Johnson Foundation-funded program that

was designed to demonstrate the feasibility and effectiveness of combining best practice treatment of depression with financial and non-financial incentives for changing systems of care. As a result of these projects and experiences, he is highly experienced with state-of-the-art techniques for dissemination of evidence-based care for mood and anxiety disorders, the conduct of mental health services research in non-psychiatric settings, and has established a network of local, national, and international collaborators. Dr. Rollman will have overall responsibility for implementing and monitoring all phases of the proposed research plan.

Bea Herbeck Belnap, PhD, Psychologist Co-Investigator Senior Research Associate, Center for Research on Health Care, University of Pittsburgh School of Medicine. Dr. Herbeck Belnap is a medical psychologist with over 13 years of research experience in medical psychology in Germany before joining Dr. Rollman’s research team in 2000, first as a member of his Robert Wood Johnson Foundation-funded Clinical Model Technical Assistance Team, then, since 2001, as project coordinator psychologist for his NIMH-funded Anxiety trials, and as senior project coordinator and study psychologist for his trial to treat post-CABG depression and CHF R34. Dr. Herbeck Belnap also assisted in the preparation of the publications from all of these studies. In Year 1 of this project, she will work closely with the Data Center to update their paperless data management system for use in this trial; conduct the CME-type in-services to educate PCPs and staff about the trial at our study practices with Dr. Rollman; train and supervise the TBH project coordinator, and help Dr. Rollman and the other co-investigators draft the study’s procedure manual. In later years of the project, she will coordinate our back-up suicidal protocol with Dr. Karp, our study psychiatrist, host chat sessions for our ISG, and participate in data analyses, manuscript presentation, and dissemination activities.

Kenneth J Smith, MD, MS, Economic Co-Investigator is Associate Professor of Medicine and Clinical and Translational Science, Section of Decision Sciences and Clinical Systems Modeling, Division of General Internal Medicine, University of Pittsburgh School of Medicine. Dr. Smith is a recognized expert in cost-effectiveness analysis, comparative effectiveness research, and the elicitation and interpretation of quality of life utility values. He has used these techniques in a wide range of clinical realms including erectile dysfunction, postherpetic neuralgia, diabetes care, and the prevention and treatment of infectious diseases. Also relevant to this application, Dr. Smith is presently collaborating with Dr. Rollman on another NIMH-funded project to evaluate various methods to pay for psychotropic medications for Medicare beneficiaries (5RC1MH088510; PI: Zhang). He will provide the study with his critical expertise by reviewing our data collection procedures in Year 1, and later, data collection, analyses, and preparation of manuscripts with respect to economic issues in Year 4.

Jordan F. Karp, MD, Psychiatrist Co-Investigator. Dr. Karp is Assistant Professor of Psychiatry, Anesthesiology, and Clinical and Translational Science at Western Psychiatric Institute and Clinic at the University of Pittsburgh School of Medicine. His NIH, RAND, and Hartford Foundation-funded research focuses on: 1) stepped care treatments for older adults with difficult to treat depression, and 2) improved treatment options for older primary care patients with comorbid depression and persistent pain. Dr. Karp is skilled at providing psychiatric care for patients living with medical illness as evidenced by his roles as Medical Director for Geriatric Psychiatry at the UPMC Pain Medicine Clinic, and as an attending physician on the psychiatry consultation-liaison service. He has authored or co-authored numerous scientific articles and book chapters, including several on treatment of medical patients with co-morbid anxiety. Dr. Karp is also expert in the provision of manualized psychotherapies such as cognitive behavioral psychotherapy, problem solving therapy, and interpersonal psychotherapy. He will provide his psychiatric expertise to the project on a regular basis through participation in the training and monitoring of our staff in use of the CCBT

program; weekly case review sessions with the study care managers providing back-up to our research staff in the event one needs to discuss a study patient’s emerging suicidality, and in analyzing data and preparing reports for dissemination of our findings.

Sati Mazumdar, PhD, Senior Statistician Co-Investigator. Dr. Mazumdar is Professor of Biostatistics, University of Pittsburgh Graduate School of Public Health at the University of Pittsburgh. She has extensive research experience in the conduct of clinical trials for treatment of mental health conditions. Since 2002, Dr. Mazumdar has collaborated with Dr. Rollman on his CABG and anxiety trials and the R34 pilot work described in this application, and she will continue to provide the key statistical expertise required for this project. In Year 1 of the proposal, she will work with Dr. Rollman and our Data Center on data management issues including establishing a file format, and other data coding procedures. During the intervention period, she will implement our randomization procedures and supervise the production of DSMB analyses and other reports with a data analyst (TBH) under her supervision. Later, Dr. Mazumdar will supervise the more complex statistical data analyses and edit the methodological aspects of the manuscripts produced.

7.2 Indicate all sources of support for this research study.

(Documents are attached in IRB form).

Federal sponsor	Grant Title	Grant number	Awardee institution	Federal grant application
NIH/NHLBI	Blended Collaborative Care for Heart Failure and Co-Morbid Depression	1R01 HL114016	University of Pittsburgh	Rollman Blended Depression-HF R01 2012.pdf(0.01)
NIH/NHLBI	Blended Collaborative Care for Heart Failure and Co-Morbid Depression	1R01HL114016	University of Pittsburgh	submission(0.01)
