# Supplementary material for: Efficacy of Blended Collaborative Care for Patients With Heart Failure and Comorbid Depression: A Randomized Clinical Trial
Source: JAMA Intern Med. 2021 Aug 30;181(10):1369–80. doi: 10.1001/jamainternmed.2021.4978 (PMC8406216; doi:10.1001/jamainternmed.2021.4978)
Supplement: Supplement 2. — Statistical Analysis Plan [file jamainternmed-e214978-s002.pdf]

Final Statistical Analysis Plan

BLENDED COLLABORATIVE CARE FOR HEART FAILURE AND CO-MORBID DEPRESSION: A RANDOMIZED CLINICAL TRIAL

Study Protocol (for further details)**:**

Herbeck Belnap B, Anderson A, Abebe KZ, et al. Blended Collaborative Care to Treat Heart Failure and Comorbid Depression: Rationale and Study Design of the Hopeful Heart Trial. *Psychosom Med.* 2019;81:495-505.

Study Design & Objectives. This a pragmatic trial whose objectives are to: (1) evaluate the effectiveness of a telephone-delivered "blended" collaborative care intervention for treating both depression and heart failure (HF) that could be readily adopted into routine clinical practice if proven effective; and (2) advance our understanding of the moderators and mediators of depression treatment on clinical outcomes. We propose to screen hospitalized adults with HF for depression and then randomize 625 protocol-eligible patients with a cardiac ejection fraction (EF) ≤ 40%, New York Heart Association (NYHA) class II-IV symptoms, and who have at least a moderately elevated level of depressive symptoms (PHQ-9 ≥ 10) at two-weeks following hospital discharge to a 12-month course of either: (1) Collaborative care for treating both HF and depression ("blended"); (2) Collaborative care for treating HF alone (enhanced usual care (eUC)); or (3) Their doctors' "usual care" for treating HF and depression (UC). Additionally, we will enroll 125 non-depressed HF patients to better evaluate the benefits derived from treating depression and, together with UC, control for any secular changes in the management of HF as elements of the 2010 Affordable Care Act are phased-in (Total N=750). Our co-primary hypotheses will test whether "blended" collaborative care can produce at 12-months follow-up a: (A) moderate 0.50 effect size (ES) or greater improvement in HRQoL vs. UC; and (B) clinically meaningful 0.30 ES or greater improvement in HRQoL vs. eUC. Our secondary hypotheses will evaluate comparisons with eUC and UC the effects of our "blended" intervention on mood,

functional status, delivery of and adherence with guideline-consistent care, incidence of cardiovascular events, health care utilization, and costs.

Sample Size Calculation. Power for this trial will be based on co-primary hypotheses that blended participants will report a clinically meaningful 0.50 12-month effect size (Cohen's d ES) improvement on the SF-12 mental component score (MCS), our primary outcome measure, versus UC (hypothesis A); and will report a 0.30 12-month ES improvement versus eUC (hypothesis B). Assuming a 80% 12-month assessment completion rate and 2-tailed type I error of 0.05, randomizing 625 participants in a 2:2:1 ratio to Blended, eUC, and UC would provide >98% power to detect a 0.50 ES improvement of Blended over UC (hypothesis A) and 85% power to detect a 0.30 ES improvement of Blended over eUC (hypothesis B) on the SF-12 MCS as well as other continuous outcome measures. Additionally, we plan to test our co-primary hypotheses within gender. Assuming a similar distribution of males and females among the randomized participants, we would have 80% power to detect an ES ≥ 0.50 (hypothesis A) and ≥ 0.40 (hypothesis B) on our primary outcome within gender.

Interim & Final Analyses. This study will not have planned interim looks for the primary outcome. The final analyses will be conducted once study follow-up is complete, after all data is cleaned, and once the study database is locked.

Hypotheses. Our co-primary hypotheses will test whether "blended" collaborative care can produce at 12-months follow-up a: (A) moderate 0.50 effect size (ES) or greater improvement in HRQoL vs. UC; and (B) clinically meaningful 0.30 ES or greater improvement in HRQoL vs. eUC. Our secondary hypotheses will evaluate comparisons with eUC and UC the effects of our "blended" intervention on mood, functional status,

delivery of and adherence with guideline-consistent care, incidence of cardiovascular events, health care utilization, and costs.

Analysis Sets. The full analysis set will be based on an intention-to-treat (ITT) analysis, which will comprise all participants who have been randomized to any of the 3 study arms, regardless of length of follow-up or actual intervention received. There will be no a priori defined per-protocol analyses.

Study Outcomes. The primary outcome is mental health-related quality of life (HRQoL) as defined by the SF-12 Mental Component Score (MCS). Secondary outcomes include: Kansas City Cardiomyopathy Questionnaire (KCCQ), Hamilton Rating Scale for Depression (HRS-D), PROMIS-Depression, SF-12 Physical Component Score (PCS), incidence of rehospitalization, all-cause and cardiovascular-related mortality, healthcare costs, and employment.

Handling of Missing Values. As a preventive measure, we will make every attempt to document all reasons for missing data. In addition, baseline characteristics will be compared between participants who do and do not withdraw from the study as a way to assess the impact of missing information and attrition. We will also compare the rates of lost-to-follow-up (LTF) between study arms. We will investigate the reasons for intermittently missing data (misses an assessment but comes back) and dropouts and use a likelihood-based procedure if "missing at random" (MAR) is confirmed or consider shared parameter models if the missingness is found to be nonignorable (missing not at random (MNAR)).

Statistical Analyses. Demographic and baseline characteristics will be presented as mean and standard deviations for continuous variables and sample proportions for categorical variables. All descriptive statistics will be accompanied by 95% confidence intervals and will be described within and across study arms. Additionally, baseline comparisons between depressed and non-depressed cohorts will be performed using t-tests for continuous variables, chi-squared tests for categorical variables, or their nonparametric counterparts.

The primary analyses will consider an intention-to-treat (ITT) analysis to describe the main findings.

Primary Outcome: The ITT analyses will assess the effect of the Blended intervention versus UC (hypothesis A) and eUC (hypothesis B), respectively, on 12-month improvements in mental health-related quality of life (HRQoL), as measured by the SF-12 MCS. We will fit a Laird and Ware linear mixed model[1] as a function of the following predictors: study arm, time, time-by-study arm, hospital type (university, community, or community underserved), and gender. In addition, the intercept will be allowed to vary randomly to account for subject-level variability of the outcome at baseline. The co-primary hypotheses will involve contrasts to estimate the adjusted mean difference in 12-month improvement on SF-12 MCS between Blended and UC (hypothesis A) as well as eUC (hypothesis B) study arms. Additionally, all analyses will be conducted separately by gender.

Secondary Outcomes: Similar to SF-12 MCS, a Laird and Ware linear mixed model will be fit to estimate the adjusted mean differences in 12-month improvement on KCCQ-12, HRS-D, PROMIS-D, and SF-12 PCS. In order to analyze time-to-first rehospitalization, all-cause and CV-related mortality, Cox proportional hazard models will be used with

study arm, hospital type, and gender as covariates; hazard ratios along with 95% confidence intervals will be calculated. Additionally, we will analyze recurrent rates of rehospitalization. All analyses will also be conducted separately by gender.

Subgroup and Exploratory Analyses: Planned subgroup analyses will be conducted for the primary outcome of SF-12 MCS. A formal hypothesis will be tested with an interaction between each of the study arms and the following subgroups: 1) gender, 2) age group, 3) race (white versus non-white), 4) education level (<= HS vs > HS), 5) social support (top quartile vs. below), 6) NYHA class (II vs III/IV), 7) baseline cardiac ejection fraction (<35% vs. >=35%), 8) etiology of HF, 9) level of mood symptoms (PHQ-9 10-14 vs. 15+), 10) history of chronic depression, 11) diabetes and other medical co-morbidity as appropriate, and 12) living alone. A significant 3-way interaction between time, study arm, and the potential covariate will indicate a subgroup effect.

To better understand how patients use the elements of our interventions and which may be most effective at improving treatment outcomes we will conduct exploratory post-hoc analyses of several measures of "dose" as a mediator of treatment effects (e.g., number of care manager contacts; HF and depression process measures of care and pharmacotherapy; PCP visits; participation in a cardiac rehabilitation program and physical activity; tobacco use; sleep quality and duration). We will use Kraemer's methodology to assess if one or more of these factors is intermediate in a causal relationship between study assignment and a dependent variable and therefore a mediator of our observed outcomes by evaluating whether the "dose": (1) is related to treatment; (2) is associated with the outcome; and (3) results in attenuation of the treatment affect after adjustment in the model.[2]

Non-Depressed Cohort Analyses: We will enroll a cohort of 125 non-depressed HF patients to facilitate comparisons with depressed patients randomized to our study interventions. Due to inherent differences between the depressed and non-depressed cohorts, any analyses conducted will need to account for potential confounding on

baseline and demographic characteristics. In order to assess differences between cohorts on the primary and secondary outcomes stated above, we will utilize propensity score methods to select groups of similar participants. Logistic regression will be used to model the propensity of being in depressed cohort as a function of baseline and demographic characteristics. Propensity score matching with a 5:1 ratio or inverse probability treatment weighting (IPTW) will be used in conjunction with linear mixed models and Cox proportional hazard models.

References:

1. Laird NM, Ware JH. Random-effects models for longitudinal data. *Biometrics*. 1982;38:963-974. doi:10.2307/2529876

2. Kraemer H, Kiernan M, Essex M, Kupfer DJ. How and Why Criteria Defining Moderators and Mediators Differ Between the Baron Kenny and MacArthur Approaches. *Heal Psychol*. 2008;27(2(Suppl.)):S101-S108.
