# Supplementary material for: Efficacy of Blended Collaborative Care for Patients With Heart Failure and Comorbid Depression: A Randomized Clinical Trial
Source: JAMA Intern Med. 2021 Aug 30;181(10):1369–80. doi: 10.1001/jamainternmed.2021.4978 (PMC8406216; doi:10.1001/jamainternmed.2021.4978)
Supplement: Supplement 3. — eFigure 1. 12-Month effect sizes for all randomized depressed patients and by gender. eFigure 2. 12-Month Readmissions: (A) All-Cause; and (B) Cardiovascular-Related. eFigure 3. 12-Month Mortality: (A) All-Cause; and (B) Cardiovascular-Related. eTable 1. Significance test for follow up rates between randomized arms and between depressed vs non-depressed. eTable 2. Readmission incidence rate per person year, All-Cause and Cardiovascular. eTable 3. Mortality incidence rate per person year, All-Cause and Cardiovascular. [file jamainternmed-e214978-s003.pdf]

Supplemental Online Content

Rollman BL, Anderson AM, Rothenberger SD, et al. Efficacy of blended collaborative care for patients with heart failure and comorbid depression: a randomized clinical trial. *JAMA Intern Med*. Published online August 30, 2021.
doi:10.1001/jamainternmed.2021.4978

eFigure 1. 12-Month effect sizes for all randomized depressed patients and by gender.

eFigure 2. 12-Month Readmissions: (A) All-Cause; and (B) Cardiovascular-Related.

eFigure 3. 12-Month Mortality: (A) All-Cause; and (B) Cardiovascular-Related.

eTable 1. Significance test for follow up rates between randomized arms and between depressed vs non-depressed.

eTable 2. Readmission incidence rate per person year, All-Cause and Cardiovascular.

eTable 3. Mortality incidence rate per person year, All-Cause and Cardiovascular.

This supplemental material has been provided by the authors to give readers additional information about their work.

© 2021 Rollman BL et al. *JAMA Internal Medicine.*

eFigure 1. 12-Month effect sizes for all randomized depressed patients and by gender.

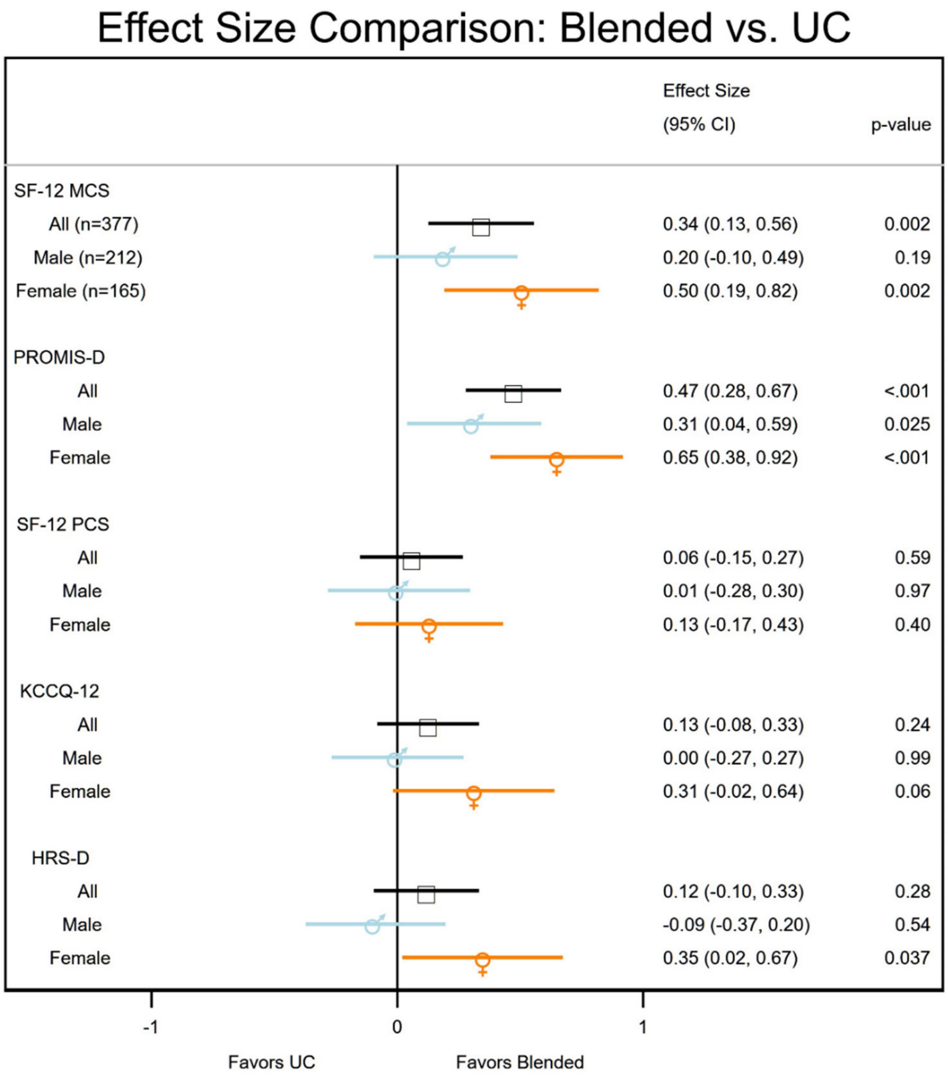

© 2021 Rollman BL et al. *JAMA Internal Medicine.*

Effect Size Comparison: Blended vs. eUC

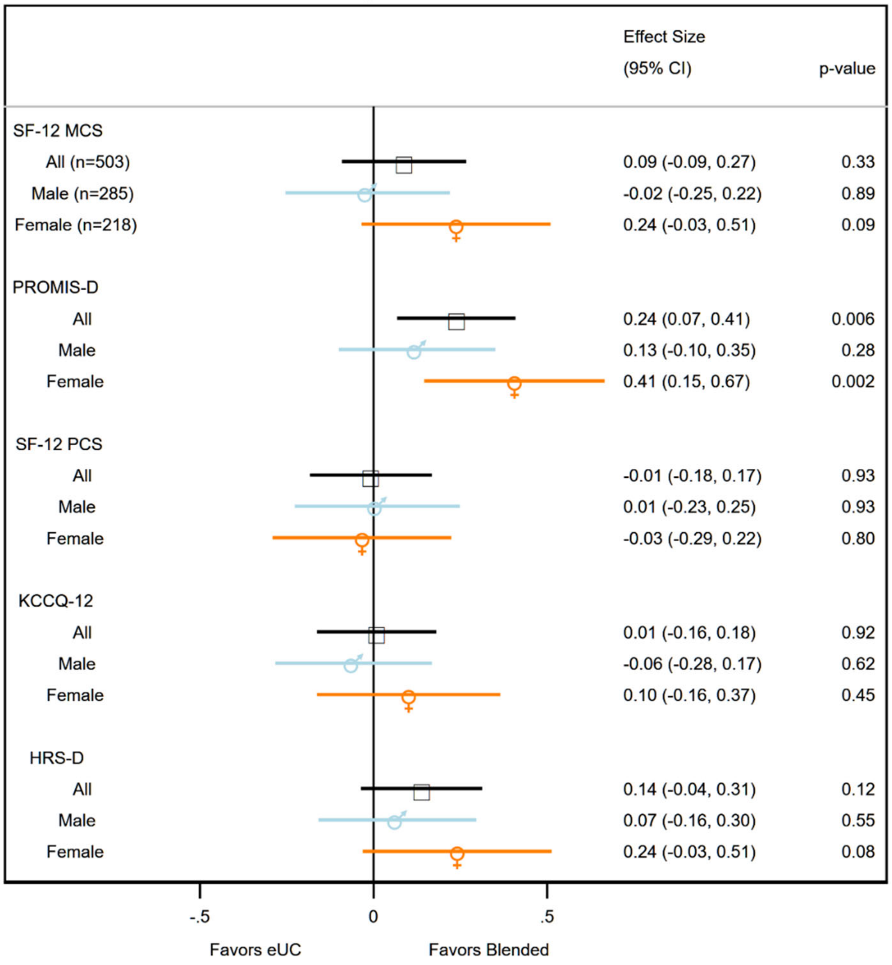

© 2021 Rollman BL et al. *JAMA Internal Medicine.*

eFigure 2. 12-Month Readmissions: (A) All-Cause; and (B) Cardiovascular-Related.

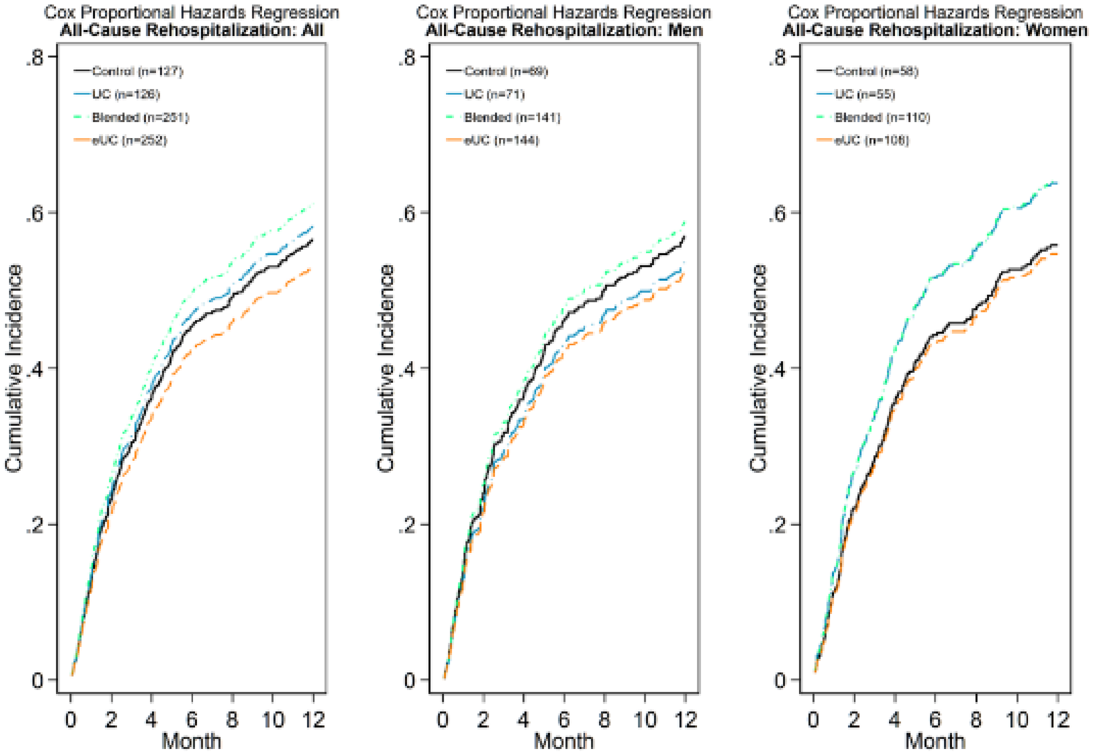

All-Cause Readmissions: Exact Incidence Rates (from above figures)

% Incidence at 12 Mo.	All	Men	Women
Blended	61.2%	58.7%	63.9%
eUC	53.1%	52.4%	54.7%
UC	58.2%	53.6%	63.8%
Non-Depressed Control	56.5%	56.9%	55.8%
P-values: Depressed Randomized Arms (UC, Blended, eUC)	0.64	0.67	0.57
P-values: All Depressed vs. Non-Depressed Controls	0.21	0.53	0.30

© 2021 Rollman BL et al. *JAMA Internal Medicine.*

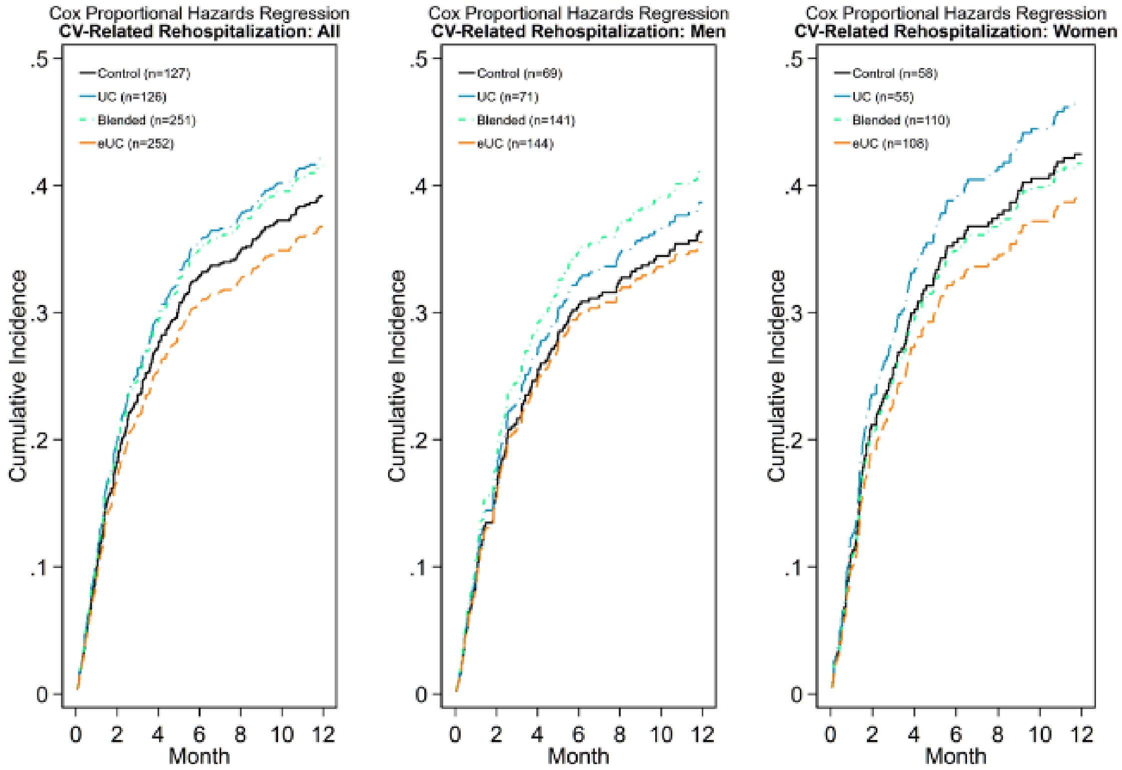

Cardiovascular Readmissions: Exact Incidence Rates (from above figures)

% Incidence at 12 Mo.	All	Men	Women
Blended	41.6%	41.2%	41.7%
eUC	36.8%	35.5%	39.0%
UC	42.2%	38.7%	46.5%
Non-Depressed Control	39.2%	36.4%	42.5%
P-values: Depressed Randomized Arms (UC, Blended, eUC)	0.85	0.79	0.75
P-values: All Depressed vs. Non-Depressed Controls	0.34	0.56	0.51

© 2021 Rollman BL et al. *JAMA Internal Medicine.*

eFigure 3. 12-Month Mortality: (A) All-Cause; and (B) Cardiovascular-Related.

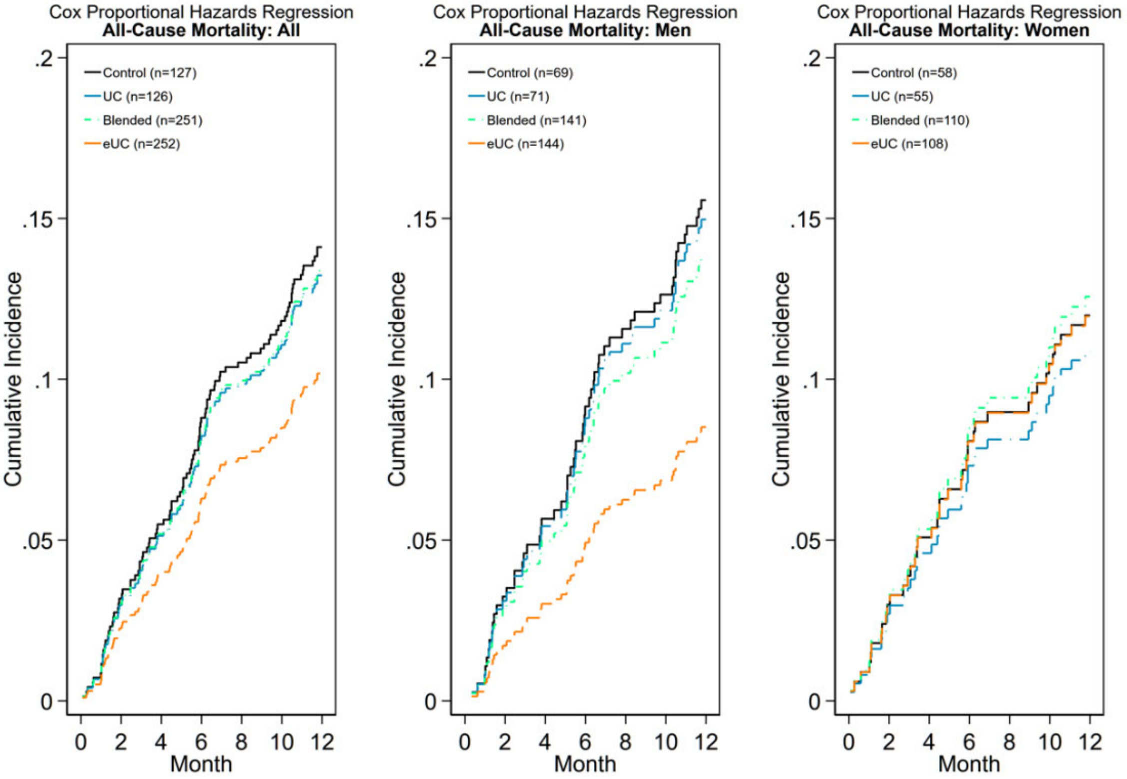

All-Cause Mortality: Exact Incidence Rates (from above figures)

**% Incidence at 12 Mo.**	**All**	**Men**	**Women**
Blended	13.4%	13.8%	12.6%
eUC	10.2%	8.5%	12.0%
UC	13.2%	15.0%	10.9%
Non-Depressed Control	14.1%	15.6%	12.0%
P-values: Depressed Randomized Arms (UC, Blended, eUC)	0.97	0.93	0.93
P-values: All Depressed vs. Non-Depressed Controls	0.31	0.17	0.97

© 2021 Rollman BL et al. *JAMA Internal Medicine.*

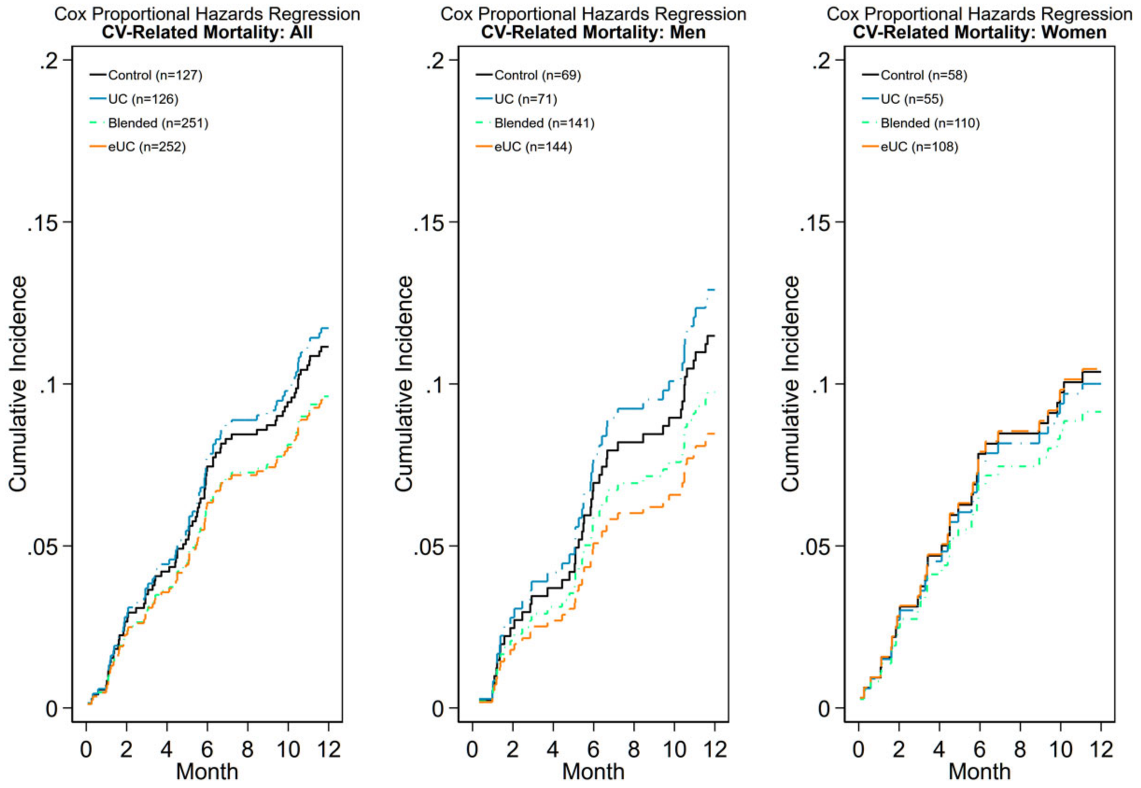

Cardiovascular Mortality: Exact Incidence Rates (from above figures)

% Incidence at 12 Mo.	All	Men	Women
Blended	9.6%	9.7%	9.1%
eUC	9.5%	8.5%	10.5%
UC	11.7%	12.9%	10.0%
Non-Depressed Control	11.1%	11.5%	10.4%
P-values: Depressed Randomized Arms (UC, Blended, eUC)	0.74	0.71	0.96
P-values: All Depressed vs. Non-Depressed Controls	0.67	0.47	0.87

© 2021 Rollman BL et al. *JAMA Internal Medicine.*

eTable 1. Significance test for follow up rates between randomized arms and between depressed vs non-depressed.

		eUC (n=252)	Blended (n=251)	UC (n=126)	P [a]	D-Randomized (n=629)	ND-Control (n=127)	P [a]
3-mo. follow-up	Completed	223	220	113	0.84	556	117	0.22
	Missed/Withdrawn	29	31	13		73	10	
6-mo. follow-up	Completed	214	205	105	0.62	524	114	0.07
	Missed/Withdrawn	38	46	21		105	13	
12-mo. follow-up	Completed	191	186	92	0.82	469	104	0.08
	Missed/Withdrawn	61	65	34		160	23	

[a] Pearson's Chi-Square Test

© 2021 Rollman BL et al. *JAMA Internal Medicine.*

eTable 2. Readmission incidence rate per person year, All-Cause and Cardiovascular.

eTable 2a. All-Cause Readmission

	eUC (N=252)	Blended Care (N=251)	Usual Care (N=126)	Randomized (N=629)	Control (N=127)	Overall (N=756)	Male, Randomized (N=356)	Female, Randomized (N=273)
**No readmission in 12-mo F/U, N (%)**	100 (40)	106 (42)	57 (45)	263 (42)	60 (47)	323 (43)	158 (44)	105 (38)
**Readmission in 12-mo F/U, N (%)**	152 (60)	145 (58)	69 (55)	366 (58)	67 (53)	433 (57)	198 (56)	168 (62)
**Incidence Rate per Person-Year,**	1.099 [0.937, 1.288]	0.993 [0.843, 1.168]	0.934 [0.738, 1.183]	1.021 [0.922, 1.132]	0.852 [0.670, 1.182]	0.991 [0.902, 1.089]	0.957 [0.833, 1.100]	1.110 [0.954, 1.291]

- Incidence rate corresponds to the time to first all-cause rehospitalization
- Within 12 months, total 1145 re-hospitalizations from 433 subjects who were ever re-hospitalized.

P-value of Randomized vs. Control = 0.220 (from Cox PH model)
P-value of Blended vs. Control = 0.341 (from Cox PH model)
P-value of Female vs. Male = 0.211 (from Cox PH model)
P-value of group effect for Men = 0.7577 (from Cox PH model)

P-value of UC vs. Control = 0.583 (from Cox PH model)
P-value of eUC vs. Control = 0.133 (from Cox PH model)
P-value of group effect for All = 0.4862 (from Cox PH model)
P-value of group effect for Women = 0.5040 (from Cox PH model

eTable 2b. Cardiovascular Readmissions

	eUC (N=252)	Blended Care (N=251)	Usual Care (N=126)	Randomized (N=629)	Control (N=127)	Overall (N=756)	Male, Randomized (N=356)	Female, Randomized (N=273)
**No CV readm in 12-mo F/U, N (%)**	149 (59)	146 (58)	78 (62)	373 (59)	81 (64)	454 (60)	218 (61)	155 (57)
**CV Readm in 12-mo F/U, N (%)**	103 (41)	105 (42)	48 (38)	256 (41)	46 (36)	302 (40)	138 (39)	118 (43)
**Incidence Rate per Person-Year**	0.617 [0.508, 0.748]	0.622 [0.513, 0.753]	0.549 [0.413, 0.728]	0.605 [0.535, 0.683]	0.512 [0.383, 0.684]	0.588 [0.526, 0.659]	0.566 [0.479, 0.669]	0.657 [0.548, 0.786]

- Incidence rate corresponds to the time to first CV-related rehospitalization

- Within 12 months, there was a total of 553 CV-related re-hospitalizations from 302 subjects.

P-value of Randomized vs. Control = 0.345 (from Cox PH model)
P-value of Blended vs. Control = 0.312 (from Cox PH model)
P-value of Female vs. Male = 0.265 (from Cox PH model)
P-value of group effect for Men = 0.845 (from Cox PH model)

P-value of UC vs. Control = 0.693 (from Cox PH model)
P-value of eUC vs. Control = 0.374 (from Cox PH model)
P-value of group effect for All = 0.748 (from Cox PH model)
P-value of group effect for Women = 0.797 (from Cox PH model

© 2021 Rollman BL et al. *JAMA Internal Medicine.*

eTable 3. Mortality incidence rate per person year, All-Cause and Cardiovascular.

eTable 3a. All-Cause Mortality

	eUC (N=252)	Blended Care (N=251)	Usual Care (N=126)	Randomized (N=629)	Control (N=127)	Overall (N=756)	Male, Randomized (N=356)	Female, Randomized (N=273)
**Alive at 12-mo F/U, N (%)**	218 (87)	218 (87)	108 (86)	544 (86)	114 (90)	658 (87)	304 (85)	240 (88)
**Deceased before 12-mo F/U, N (%)**	34 (13)	33 (13)	18 (14)	85 (14)	13 (10)	98 (13)	52 (15)	33 (12)
**Incidence Rate per Person-Year**	0.145 [0.104, 0.203]	0.143 [0.102, 0.201]	0.153 [0.097, 0.243]	0.146 [0.118, 0.187]	0.109 [0.063, 0.187]	0.136 [0.114, 0.170]	0.158 [0.121, 0.208]	0.130 [0.092, 0.182]

- Incidence rate corresponds to the time to all-cause mortality

P-value of Randomized vs. Control = 0.321 (from Cox PH model)
P-value of Blended vs. Control = 0.397 (from Cox PH model)
P-value of Female vs. Male = 0.592 (from Cox PH model)
P-value of group effect for Men = 0.5962 (from Cox PH model)
P-value of UC vs. Control = 0.341 (from Cox PH model)
P-value of eUC vs. Control = 0.377 (from Cox PH model)
P-value of group effect for All = 0.7898 (from Cox PH model)
P-value of group effect for Women = 0.9840 (from Cox PH model)

eTable 3b. Cardiovascular Mortality

	eUC (N=252)	Blended Care (N=251)	Usual Care (N=126)	Randomized (N=629)	Control (N=127)	Overall (N=756)	Male, Randomized (N=356)	Female, Randomized (N=273)
**Alive at 12-mo F/U, N (%)**	228 (90)	222 (88)	112 (89)	562 (89)	115 (91)	677 (90)	316 (89)	246 (90)
**Deceased before 12-mo F/U, N (%)**	24 (10)	29 (12)	14 (11)	67 (11)	12 (9)	79 (10)	40 (11)	27 (10)
**Incidence Rate per Person-Year**	0.102 [0.069, 0.153]	0.126 [0.087, 0.181]	0.119 [0.071, 0.201]	0.115 [0.090, 0.146]	0.100 [0.057, 0.177]	0.112 [0.090, 0.140]	0.122 [0.089, 0.166]	0.106 [0.073, 0.155]

- Incidence rate corresponds to the time to CV-related mortality

P-value of Randomized vs. Control = 0.678 (from Cox PH model)
P-value of Blended vs. Control = 0.522 (from Cox PH model)
P-value of Female vs. Male = 0.709 (from Cox PH model)
P-value of group effect for Men = 0.7491 (from Cox PH model)
P-value of UC vs. Control = 0.671 (from Cox PH model)
P-value of eUC vs. Control = 0.974 (from Cox PH model)
P-value of group effect for All = 0.8543 (from Cox PH model)
P-value of group effect for Women = 0.9910 (from Cox PH model)

© 2021 Rollman BL et al. *JAMA Internal Medicine.*
