# Supplementary material for: Efficacy of Blended Collaborative Care for Patients With Heart Failure and Comorbid Depression: A Randomized Clinical Trial
Source: JAMA Intern Med. 2021 Aug 30;181(10):1369–80. doi: 10.1001/jamainternmed.2021.4978 (PMC8406216; doi:10.1001/jamainternmed.2021.4978)
Supplement: Supplement 4. — Data Sharing Statement [file jamainternmed-e214978-s004.pdf]

Data Sharing Statement

Rollman. Efficacy of Blended Collaborative Care for Patients With Heart Failure and Comorbid Depression. *JAMA Intern Med*. Published August 30, 2021. doi:10.1001/jamainternmed.2021.4978

Data
Data available: Yes
Data types: Deidentified participant data, Data dictionary
How to access data: Requests can be sent to the PI: rollmanbl@upmc.edu and to the NHLBI BioLINCC Data Repository: https://biolincc.nhlbi.nih.gov/home/
When available: With publication

Supporting Documents
Document types: Statistical/analytic code
How to access documents: Requests can be sent to the PI: rollmanbl@upmc.edu and to the NHLBI BioLINCC Data Repository: https://biolincc.nhlbi.nih.gov/home/
When available: With publication

Additional Information
Who can access the data: Who can access the data: Researchers whose proposed use of data has been approved and with a signed data use agreement (DUA).
Types of analyses: For any purpose.
Mechanisms of data availability: With a signed data use agreement (DUA) either from the investigators or without investigator support through NHLBI BioLINCC.
